# Bitter and sweet taste perception: relationships to self-reported oral hygiene habits and oral health status in a survey of Australian adults

**DOI:** 10.1186/s12903-021-01910-8

**Published:** 2021-10-29

**Authors:** Kiranjit Kaur, Dean Sculley, Martin Veysey, Mark Lucock, Janet Wallace, Emma L. Beckett

**Affiliations:** 1 School of Environmental and Life Sciences, The University of Newcastle, Ourimbah, NSW Australia; 2 School of Biomedical Sciences and Pharmacy, The University of Newcastle, Ourimbah, NSW Australia; 3 School of Medicine and Public Health, The University of Newcastle, Ourimbah, NSW Australia; 4 School of Health Sciences, The University of Newcastle, Ourimbah, NSW Australia; 5 Hunter Medical Research Institute, New Lambton Heights, NSW Australia; 6 Priority Research Centre for Physical Activity and Nutrition, The University of Newcastle, Ourimbah, NSW Australia

**Keywords:** Bitter, Sweet, Taste, Oral health, Oral hygiene, Perception, Intensity, Liking

## Abstract

**Background:**

Oral health, an essential part of general health and well-being, is influenced by multiple factors, including oral hygiene habits and dietary factors. Dietary preferences are influenced by variation in taste perceptions and threshold tasting. Polymorphisms in specific genes for sweet and bitter taste receptors and bitter taste perception have been associated with dental caries. However, taste is complex with multiple receptors, each with multiple potential polymorphisms contributing to taste perception as well as social, cultural, and environmental influences. Additionally, these association studies have been conducted in restricted cohorts (e.g., children only). Furthermore, outcomes have been limited to dental caries and studies between taste perception and oral hygiene habits have not been completed.

**Methods:**

A cross-sectional online survey was conducted to investigate the relationships between bitter and sweet taste perception (liking and intensity of index food items), self-reported oral hygiene habits and oral health (n = 518).

**Results:**

Higher mean intensity scores for bitter (16–21%) and sweet (< 5%-60%) were seen with higher frequencies of oral hygiene habits (brushing, use of mouthwash, chewing gum and tongue cleaning). Lower mean bitter liking scores (18–21%) were seen with higher frequencies of oral hygiene habits (brushing, mouthwash use, floss use and chewing gum). Sweet liking scores varied by reported frequency of mouthwash use and flossing only, with mixed patterns of variance. Mean bitter and sweet intensity perception scores varied with the number of dental caries ((13–20% higher in those with 3 or more caries, compared to none).

**Conclusions:**

While there were numerous relationships identified between liking and perception of sweet and bitter and oral health outcomes, the magnitude and direction of associations varied by outcome. The direction of the associations cannot be inferred due to the cross-sectional nature of the study. The demonstrated relationships justify further future investigations, which could help better understand if taste liking and perception is impacted by oral hygiene and health, or vice versa. This could be important in understanding the causation and progression of oral health diseases or the development of novel therapeutics for oral health.

**Supplementary Information:**

The online version contains supplementary material available at 10.1186/s12903-021-01910-8.

## Background

Oral health is an essential part of general well-being [1, 2]. Oral diseases have numerous negative impacts on daily living due to pain, infection, inflammation, and tooth loss. [3]. The number of missing natural teeth has been directly associated with the inability to eat a balanced diet resulting in lower dietary quality [4]. In 2015, poor oral health caused by tooth decay, gum diseases and tooth loss contributed to 4.5% of the non-fatal burden of diseases in Australia; affecting both children and adults [5]. Chronic, untreated oral diseases leading to tooth loss can cause reduced quality of life [3, 6] and tooth loss has also been associated with lower dietary intake and reduced diet quality [4]. The consequences of oral disease are not limited to the mouth, and they can also affect systemic health. Oral health and systemic health are closely related to each other [2] inflammatory oral diseases have been associated with coronary heart diseases and cardiovascular diseases, diabetes and dementia [7–10]. Oral diseases are multifactorial, with numerous modifiable and non-modifiable risk factors associated with oral diseases [11–13]. These factors include oral hygiene habits, smoking, ageing, systemic diseases, type of microorganisms, genetic factors, behavioural and psychological factors, and dietary factors [14–18].

Dietary factors, including intakes, are modifiable and are influenced by taste preferences [19]. It is well-established that sweet taste receptor genetics influence preference for sugars [20–22], known to influence oral health outcomes [23, 24]. Bitter taste receptor genetics are related to consumption of alcohol [25] which may impact oral health [26] and oral microbial populations [27]. Bitter taste sensitivity has also previously been linked to modulation of consumption of healthy bitter vegetables, which contain high levels of nutrients and antioxidants [19]. Sweet and bitter taste receptors have also recently been identified to have non-gustatory roles, involvement in the modulation of inflammatory responses, including in the upper respiratory tract [28, 29]; this may further influence oral health.

Most research on the relationship between taste perception and oral health has involved specific sweet and bitter taste genes and dental caries [30–32]. In a small study investigating multiple genotypes, allelic variation in sweet taste genes, including *GNAT3, SLC2A2, SLC2A4, TAS1R1* and *TAS1R2* was associated with dental caries [30]. Likewise, sweet taste sensitivity and liking associated with polymorphisms present in *TAS1R2* and *GLUT2* sweet taste genes have also been related to the higher prevalence of dental caries [32]. In a larger study considering fewer genotypes, caries status varied with *TAS2R38* and *TAS1R2* gene polymorphism status [31]. Furthermore, children who are 6-*n*-propylthiouracil (PROP) non-tasters are more prone to dental caries than tasters [33, 34]. However, these investigations were restricted to oral health outcomes without assessing oral hygiene habits, and research has been limited to specific populations.

It could also be hypothesized that oral hygiene habits may also be influenced by taste perceptions, however, this has not been investigated. Those with more sensitive tastes may have increased motivation for hygiene practices, or may be deterred to use hygiene products with intense tastes. Furthermore, oral hygiene habits may influence taste perception, with bacterial coatings on the tongue or practices such as mouthwash and tongue cleaning potentially reducing perceptions. Alternatively, perception may deter from the use of intense tasting oral hygiene products such as mouthwashes. Through these pathways, there is potential for taste variance to indirectly impact oral health outcomes.

To our knowledge, there are no data examining the relationships between perception (liking and intensity) of sweet and bitter and oral health outcomes, other than caries, nor are there data relating to oral hygiene habits. A better understanding of these relationships may be useful in identifying at-risk populations and behaviours and may assist in the design of novel targeted therapeutics. Therefore, this current exploratory study investigates the relationship between perception (liking and intensity) of sweet and bitter index foods, oral hygiene habits and oral health outcomes simultaneously, using a cross-sectional survey design.

## Methods

### Questionnaire

An online cross-sectional survey using a snowball recruitment technique was conducted. Approval was obtained from The Human Research Ethics Committee of the University of Newcastle (Reference Number: H-2020-0312), all methods were carried out in accordance with relevant guidelines and regulations. The survey was generated and managed using Qualtrics (SAP, USA). The researchers recruited participants via social media platforms from 22nd September 2020 to 18th November 2020. The inclusion criteria for participation included living in Australia, aged over 18 years with internet access and sufficient English language proficiency to complete the survey. Participants gave their informed consent before initiating the survey. To ensure low-quality responses were excluded, surveys completed in less than half the median time to complete were excluded as this was deemed unlikely to allow sufficient time for the participant to have read and understood the questions. The survey included a total of 29 questions (The complete survey is presented in Additional file 1: S1). Questions were presented in four blocks: oral health questions (self-reported oral hygiene habits and oral health), bitter and sweet taste perception scores (liking and intensity), dietary habits and demographics.

Questions on oral hygiene habits included frequency of brushing, use of mouthwash, flossing, chewing gum, and tongue cleaning, with common frequencies presented for participants to select from. Self-reported oral health outcomes included the number of missing, loose or filled teeth (selected from categories) and frequency of symptoms such as toothache, dry mouth, and bad breath. These variables were adapted from previous surveys on self-reported oral health status and oral hygiene habits [35–37].

Taste perceptions were assessed using General Labelled Magnitude Scales (GLMS) to rate liking and intensity of the sweetness and bitterness of indicator foods. The lists of foods included were taken from Cornelis et al*.* [38]*,* who identified essential foods correlated with specific taste preferences and perceptions, with minor adaptations of food names to suit the Australian cohort. Participants were asked to rate liking on a scale from 0 (most disliked imaginable) to 100 (most liked imaginable) with intermediates (‘dislike extremely, ‘dislike very much’, dislike moderately, ‘dislike slightly’, ‘neutral’, ‘like slightly’, ‘like moderately’, ‘like very much’ and ‘like extremely’) spaced at even intervals between the extremes. For intensity, the food items were rated on a scale from 0 (barely detectable)-100 (strongest imaginable). Internal labels were included at equal intervals between extremes endpoints (‘weak’, ‘moderate’, ‘strong’, ‘very strong’). Participants had the option to skip the rating by ticking ‘not applicable’ button if allergic or unfamiliar with any food item [38].

Self-reported dietary habits were assessed using a validated questionnaire used in previous studies [39]. For each question, there were 3 options with the healthiest choice scored as 3, and the least healthful choice scored as 1. The dietary index was calculated by adding these scores for all questions. Diversity scores for the intake of fruits and vegetable were evaluated using previously validated questionnaires [40, 41]. Each question was aimed to assess the intake of fruits and vegetables commonly consumed in Australia as per the previously validated Australian Healthy Eating Quiz. Participants were asked to state the fruit and vegetable intake as ‘once per week or more often’ and ‘less than once per week or never’. The total diversity scores were calculated by adding scores for once per week or more [42]. Participants were asked to report key demographic variables, including their year of birth, sex, education level, household income, weight, height (used to calculate body mass index (BMI)), and smoking history.

### Statistical analysis

Analyses were performed using JMP (Pro 14, SAS Institute Inc., Cary, NC, USA) and GraphPad prism (v9.0, GraphPad Software Inc, CA, USA). Age, BMI, diet index, taste intensity and liking were treated as continuous variables, with minimum and maximum ranges, mean, standard deviations (SD) and 95% confidence intervals presented as appropriate. Sex, income, education, smoking habits, oral hygiene habits and oral health outcomes were treated as categorical variables. Categorical variables, and descriptive statistics for categorical variables are presented as numbers and percentage. The statistically significant threshold for *p* value was < 0.05, however all p-values are displayed for transparency. Least squares means were compared using least squares regression with Tukey HSD’s post hoc test. Analyses were conducted unadjusted and adjusted for potential confounders (minimally adjusted model = age, sex and smoking status; fully adjusted model = age, sex, smoking status, income, education and dietary index; see Additional file 1: Figure S1 for potential relationships between variables). Least square mean values with 95% confidence intervals were reported. The adjustments were selected as individual factors that may impact oral health outcomes [17, 43–47]. Cronbachs alpha was applied to scales to assess internal validity [48].

## Results

A total of 621 participants provided implied consent (via checking the box “I consent that my answers can be used for research purposes” following the information statement) and commenced the survey. Ninety-eight responses were excluded due to incomplete surveys, and a further five more responses were excluded for completing too quickly (< 280 s or half the median time for completion). Therefore, a total of 518 participants were included in the analyses.

Participants were aged between 18 and 79 years (mean = 40.3 ± 13.3; Table 1). Demographic variables (sex, income, education, smoking status, and BMI) are presented in Table 1. The majority of participants reported income in the highest bracket > $150,000 with education level at a bachelor’s degree (Table 1). Due to the small numbers of participants in the lower and middle-income brackets, the categories were collapsed into ≤ $74,999 group for analysis. Similarly, the groups were also collapsed for education levels to ≤ year 12 or equivalent due to fewer responses in the categories < year 12 or equivalent and year 12 or equivalent. The smoking status variable was also collapsed into “never” and “ever” groups, with ever including those who currently smoke and those who formerly smoke, due to the low rates of smoking in respondents. BMI had a balanced distribution across categories (normal, overweight and obese), but have a skewed continuous distribution with a larger variance in BMI in the obese group. The median BMI was 26.9 (range 18.2–57.5).

**Table 1:** Demographic variables (n = 518)

Variables	Distribution (n, %)
*Sex*	
M	167 (32.2%)
F	343 (66.2%)
Non-binary	8 (1.5%)
*Income*	
≤ $74,999	109 (21.0%)
$75,000–$99,999	85 (16.4%)
$100,000–$149,999	131 (25.3%)
> $150,000	140 (27.0%)
Others	53 (10.2%)
*Education*	
≤ year 12 or equivalent	86 (16.6%)
TAFE or Technical diploma	77 (14.9%)
Bachelor’s degree	179 (34.6%)
Postgraduate degree	174 (33.6%)
Others	2 (0.0%)
*Smoking status*	
Ever	124 (23.9%)
Never	394 (76.1%)
*BMI status*	
Normal	193 (37.4%)
Overweight	132 (25.6%)
Obese	191 (37.0%)

The diet index, intensity and perception scores were relatively normally distributed. The mean dietary index score was 16.5 ± 1.9; Table 1. The mean bitter liking average score was 58.6 ± 19.0; the mean sweet liking average score was 62.9 ± 19.0. The mean bitter intensity average score was 48.9 ± 20.0, and the mean sweet intensity average score was 72.7 ± 16.2. The mean sum of fruits and vegetables diversity scores were 5.1 ± 2.6 and 8.8 ± 2.8, respectively. The Cronbachs Alpha values for all liking and intensity scales were above 0.7.

The majority of the participants (59.5%) reported brushing their teeth twice daily, and 34.4% used electronic toothbrush (Table 2). 59.5% of participants reported usage of floss for interdental cleaning. The majority of additional oral habits were reported less than weekly or never, including mouthwash (60.0%), use of floss/water pik or piksters (40.5%), chewing gum (71.4%), tongue cleaning (44.0%; Table 2). Flossing was combined with any flossing (floss, piksters and water pik) due to the low frequency of use for each method. The frequency of dental visit for check-up and cleaning was reported was once yearly (35.9% and 36.7%, respectively; Table 2).

**Table 2:** Frequencies of self-reported oral hygiene habits and oral health status

Oral hygiene habits		Oral health status markers	
	(n, %)		(n, %)
*Brushing habits*		*Missing teeth*	
≤ Weekly	18 (3.5%)	1	72 (13.9%)
1 × a day	154 (29.7%)	2 or 3	39 (7.5%)
2 × a day	308 (59.5%)	More than 3	32 (6.2%)
> 2 × a day	38 (7.3%)		
*Type of toothbrush*		*Dental caries*	
Electronic	178 (34.4%)	1	68 (13.1%)
Manual, extra soft	32 (6.2%)	2 or 3	118 (22.8%)
Manual, firm	25 (4.8%)	More than 3	189 (36.5%)
Manual, medium	123 (23.7%)		
Manual, soft	153 (29.5%)		
Others	7 (1.4%)		
*Mouthwash use*		*Toothache*	
< weekly/never	313 (60.0%)	Previously, but not in last 12 months	158 (30.1%)
Weekly	80 (15.4%)	Sometimes	125 (24.1%)
1 × a day	69 (13.3%)	Never	235 (45.4%)
2 × a day	56 (10.8%)		
*Combined frequency use of floss*		*Bad breath*	
< weekly/never	210 (40.5%)	Don’t answer	37 (7.1%)
Weekly	152 (29.3%)	No	149 (28.8%)
1 × a day	114 (22.0%)	Yes, but infrequently	287 (55.4%)
≥ 2 × a day	42 (8.1%)	Yes, regularly	45 (8.7%)
*Use of chewing-gum*		*Bleeding (yes)*	247 (47.7%)
< weekly/never	370 (71.4%)		
Weekly	50 (9.7%)		
1 × a day	53 (10.2%)		
2 × a day	45 (8.7%)		
*Tongue cleaning*		*Dry mouth*	
< weekly/never	228 (44.0%)	I don’t know	38 (7.3%)
Weekly	69 (13.3%)	No	282 (54.4%)
1 × a day	109 (21.0%)	Yes, but infrequently	139 (26.8%)
2 × a day	112 (21.6%)	Yes, regularly	59 (11.4%)
*Frequency of dental visit*			
> 1 × a year	127 (24.5%)		
1 × a year	186 (35.9%)		
≤ 1 × few years	122 (23.6%)		
Occasionally*	83 (16.0%)		
*Dental visit for cleaning*			
> 1 × a year	120 (23.2%)		
1 × a year	190 (36.7%)		
≤ 1 × few years	122 (23.6%)		
Occasionally*	86 (16.6%)		

Occasionally*-dental visit only when experienced pain or needed

27.6% of participants reported having one or more missing teeth, with 13.9% reporting only one missing tooth (Table 2). The majority of participants (36.5%) reported having more than three decayed teeth or filled teeth. 45.4% reported never experiencing toothache problems, and 30.1% reported previous toothache experience but not in the last 12 months (Table 2). More than half (55.4%) reported bad breath infrequently, and 47.7% of participants reported bleeding (Table 2). The variable regarding the frequency of bleeding from gums was collapsed into a binary variable (Yes/No) due to the low frequency of reported bleeding. The majority of participants (54.4%) reported not experiencing dry mouth (Table 2).

### The reported intensity of bitter and sweet by frequency of oral hygiene habits

**Fig. 1:**
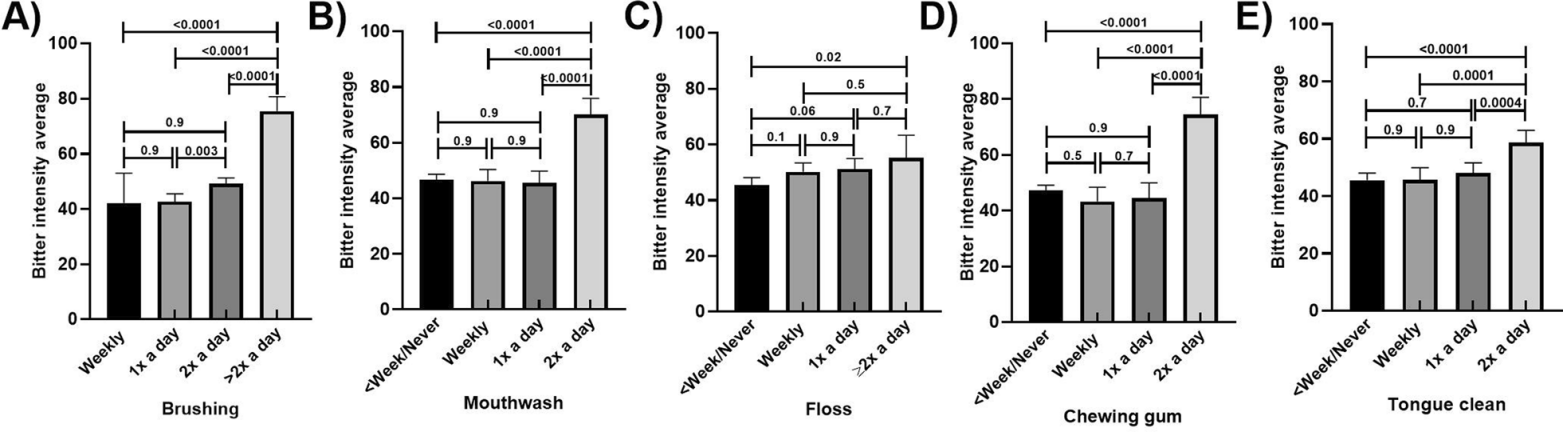
Bitter intensity scores by frequency of oral hygiene habits, unadjusted means; Frequency of **A** Brushing **B** Mouthwash use **C** Flossing **D** Chewing gum **E** Tongue Cleaning. *P* values are marked for differences between groups; Error bars mark 95% confidence intervals

Those who reported brushing and using mouthwash more regularly had higher bitter intensity scores than those who engaged in these behaviours less regularly (Fig. 1A, B, respectively). There was a 16% increase in bitter intensity scores from those who reported brushing twice a day, compared to those who brushed only twice a day, and a 43% increase from twice daily to more regularly. Those who reported using mouthwash twice a day had bitter intensity scores approximately 50% higher than those who used mouth was less regularly. These results remained similar in the adjusted models (Additional file 1: Tables S1–S4.)

Those who reported using any flossing at a frequency of twice daily (dental floss, piksters or water pik) had higher bitter intensity scores than those who flossed less than weekly or never (*p* value *p* = 0.001; 22% increase; Fig. 1C). The mean intensity score for those who chewed gum twice a day had bitter intensity scores more than 60% higher than the means for the lower frequency categories (Fig. 1D). Those who reported regular tongue cleaning (twice a day) had higher mean bitter intensity scores than those who reported engaging in togue cleaning less regularly by approximately 28% (Fig. 1E). These results remained similar in the adjusted models (Additional file 1: Tables S1–S4).

**Fig. 2:**
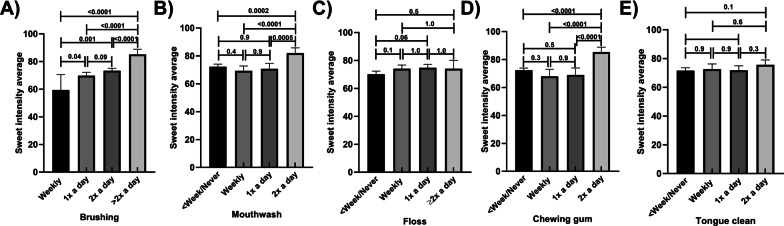
Sweet intensity scores by frequency of oral hygiene habits, unadjusted means; Frequency of **A** Brushing **B** Mouthwash use **C** Flossing **D** Chewing gum **E** Tongue Cleaning. *p* values are marked for differences between groups; Error bars mark 95% confidence intervals

Those who reported regular brushing and mouthwash use had higher sweet intensity scores than those who engaged in these activities less frequently (Fig. 2A, B, respectively). There were incremental increases in sweet intensity scores with higher frequency of brushing, with scores in the highest frequency group more than 60% higher than the lowest frequency group. Sweet intensity scores in those who reported using mouthwash twice a day were approximately 17% higher than in all other frequency groups. These results remained similar in the adjusted models (Additional file 1: Tables S5–S8). Mean sweet intensity scores did not varied by flossing habits (Fig. 2C), with similar scores across all frequency groups. Those who chewed gum more regularly had higher sweet intensity scores than those who were less frequently involved in this behaviour (Fig. 2D), with approximately 17% higher scores in those who reported the most frequent use compared to the other groups. No associations were found between frequency of tongue cleaning and sweet intensity scores (Fig. 2E). These results remained similar in the adjusted models (Additional file 1: Tables S5–S8), with the exception of those who were engaged in regular tongue cleaning habits had higher sweet intensity scores than who were less frequently involved in regular tongue cleaning, but only a small difference (< 5%) between the highest and lowest frequencies being seen (Additional file 1: Tables S5–S6.)

### The reported intensity of bitter and sweet by self-reported oral health status

**Fig. 3:**
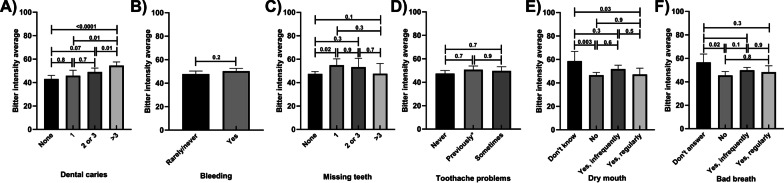
Bitter intensity scores by self-reported oral health status, unadjusted means; **A** Dental Caries **B** Bleeding **C** Missing Teeth **D** Tooth Ache Problems **E** Dry Mouth **F** Bad Breath. *p* values are marked for differences between groups; toothache problems—previously means toothache problems reported but not in last 12 months; Error bars mark 95% confidence intervals

Those who reported having 3 or more caries had higher mean bitter intensity scores than those who had one or no reported dental caries (25% and 20% increases, respectively; Fig. 3A). Bitter intensity scores did not vary between those reporting bleeding of the gums and those who did not (Fig. 3B). These results did not vary when adjusted for potential confounders. Those who reported one missing tooth had 15% higher bitter intensity scores than those who had no missing teeth (Fig. 3C). Bitter intensity scores were similar regardless of whether or not participants reported suffering from toothaches (Fig. 3D). There was no difference in bitter taste intensity ratings between those who did and did not experience dry mouth and bad breath (Fig. 3E, F, respectively). These results remained similar in the adjusted models (Additional file 1: Tables S9–S12).

**Fig. 4:**
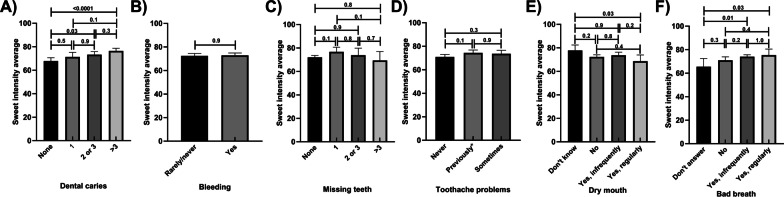
Sweet intensity scores by self-reported oral health status, unadjusted means; **A** Dental Caries **B** Bleeding **C** Missing Teeth **D** Tooth Ache Problems **E** Dry Mouth **F** Bad Breath. *p* values are marked for differences between groups; toothache problems—previously means toothache problems reported but not in last 12 months; Error bars mark 95% confidence intervals

Those who reported more than 3 dental caries had 13% higher sweet intensity scores than those who reported having no dental caries (Fig. 4A). Sweet intensity scores did not vary by reports of bleeding, missing teeth and toothache problems (Fig. 4B–D; respectively). No variations in the results were shown following the adjustment for potential confounders. Mean sweet intensity scores were related to reported frequency of experiencing dry mouth or bad breath (Fig. 4E, F). These results remained similar in the adjusted models (Additional file 1: Tables S9–S12).

### Reported liking of bitter and by frequency of oral hygiene habits

**Fig. 5:**
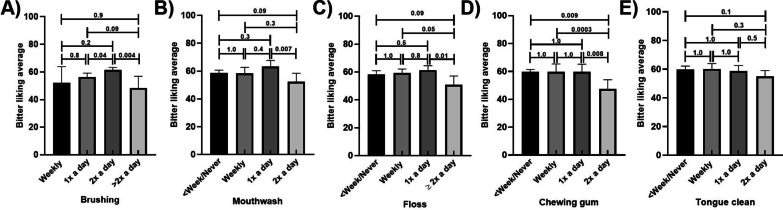
Bitter liking scores by frequency of oral hygiene habits, unadjusted means; Frequency of **A** Brushing **B** Mouthwash use **C** Flossing **D** Chewing gum **E** Tongue Cleaning. *p* values are marked for differences between groups; Error bars mark 95% confidence intervals

Those who reported regular brushing, using mouthwash, flossing, and chewing gum had lower bitter liking scores than those who engaged less frequently in these behaviours (Fig. 5A–D; respectively). Mean bitter liking scores were 8% higher in those who reported brushing twice a day compared to those who reported brushing daily, but those who brushed more than twice a day had lower scores by more than 21% (Fig. 5A). Those who reported using mouthwash or flossing twice a day has lower bitter liking scores than those who reported using mouthwash once a day, by approximately 18% for each habit (Fig. 5B, C). Those who reported chewing gum twice a day had approximately 18% lower mean bitter liking scores than all other categories of usage frequency (Fig. 5D). No relationships were found between tongue cleaning and bitter liking scores (Fig. 5E). These results remained similar in the adjusted models (Additional file 1: Tables S13–S16.)

**Fig. 6:**
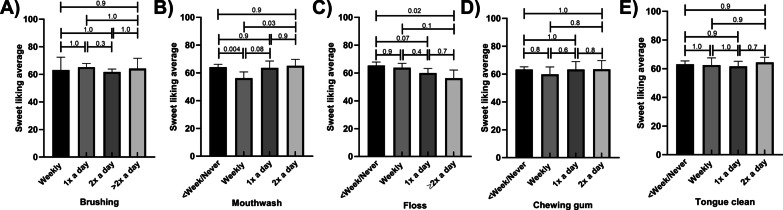
Sweet liking scores by oral hygiene habits, unadjusted means; Frequency of **A** brushing **B** Mouthwash use **C** Flossing **D** Chewing gum **E** Tongue Cleaning. *p* values are marked for differences between groups; Error bars mark 95% confidence intervals

Sweet liking scores did not vary by frequency of reported brushing (Fig. 6A). Sweet liking scores varied by frequency of mouthwash use, with mean scores higher in those who never or less than weekly used mouth wash being approximately 14% higher than those who reported using it weekly. However, this trend was not uniform, with those who reported using mouth wash twice daily having similar sweet liking scores to those who reported never using (Fig. 6B). These results remained similar in the adjusted models (Additional file 1: Tables S17–S20.) There was a trend toward lower mean intensity scores with higher frequencies of floss use (reducing by 14% from the least frequent to the most frequent users, however this was not apparent in the adjusted models (Additional file 1: Tables S17–S20). Sweet intensity scores did not vary by frequency of chewing gum and tongue cleaning (Fig. 6D, E, respectively). These results remained similar in the adjusted models (Additional file 1: Tables S17–S20.)

### Reported bitter and sweet liking by self-reported oral health status

**Fig. 7:**
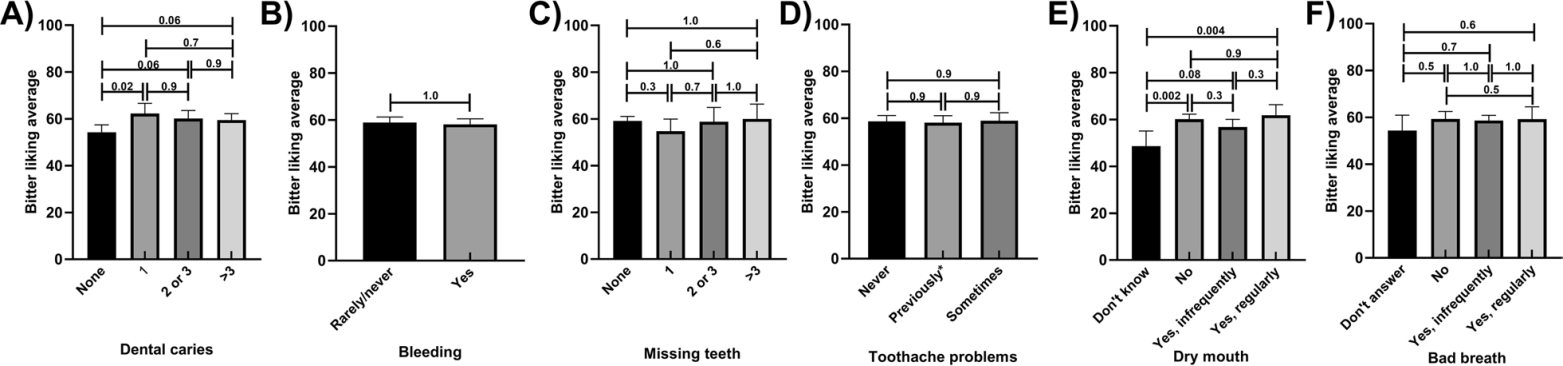
Bitter liking scores by self-reported oral health status, unadjusted means; **A** Dental Caries **B** Bleeding **C** Missing Teeth **D** Tooth Ache Problems **E** Dry Mouth **F **Bad Breath. *p* values are marked for differences between groups; toothache problems—previously means toothache problems reported but not in last 12 months; Error bars mark 95% confidence intervals

Those who reported one tooth with dental caries had approximately 15% higher mean bitter liking score than those who had none. However, there were no differences I those with more teeth with caries (Fig. 7A). Mean bitter liking scores did not vary by reported bleeding, missing teeth, toothache problems, dry mouth or bad breath (Fig. 7B–F). These results remained similar in the adjusted models (Additional file 1: Tables S21–S24.)

**Fig. 8:**
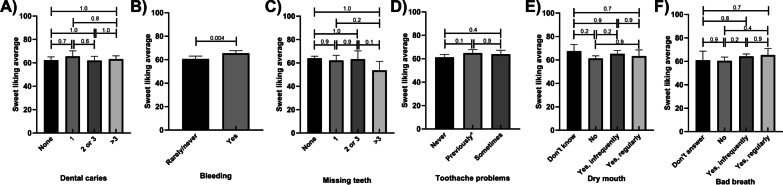
Sweet liking scores by self-reported oral health status, unadjusted means; **A** Dental Caries **B** Bleeding **C** Missing Teeth **D** Tooth Ache Problems **E** Dry Mouth **F** Bad Breath. *p* values are marked for differences between groups; toothache problems—previously means toothache problems reported but not in last 12 months; Error bars mark 95% confidence intervals

Those who reported bleeding had higher mean sweet liking scores (8%) than who reported no bleeding (Fig. 8B). Those who reported more than 3 missing teeth had lower mean sweet liking scores (16%; Fig. 8C). These scores did not vary by number of missing teeth when adjusted for potential confounders. Mean sweet liking scores did not vary by category reported dental caries, toothache problems, dry mouth, bad breath, and sweet liking scores (Fig. 8A, D–F; respectively). These results remained similar in the adjusted models (Additional file 1: Tables S29–S32.) Those who reported toothache problems previously (but not in the last 12 months) slightly higher mean sweet liking scores when adjusted for potential confounders. These results remained similar in the adjusted models (Additional file 1: Tables S28–S32.)

## Discussion

This study simultaneously explored the variation of bitter and sweet perception (liking and intensity) with self-reported oral hygiene habits and oral health outcomes. Bitter and sweet intensity scores had clear patterns of variance by frequency of key oral hygiene habits. Generally, higher mean intensity scores were seen in those who reported engaging in oral hygiene habits more frequently. Bitter liking scores also showed patterns of variance by frequency of oral hygiene habits, with lower mean scores seen in those who practice oral hygiene habits more frequently. Bitter and sweet intensity scores both varied by caries status; however no other clear patterns for self-reported oral health status and bitter or sweet taste perception were seen in these data.

The pattern of higher intensity scores with more frequent oral hygiene habits for both bitter and sweet taste modalities may support the hypothesis that more regular hygiene habits increase taste perception. This may occur through increased exposure of taste bud to tastants through reduction of bacterial coating or modifications to saliva. The finding presented here reflect previous findings in a hospitalized elderly cohort (n = 174) where high plaque scores were associated with reduced overall taste scores [49]. Although the causative direction of this association cannot be identified here, it is well-established that good oral hygiene habits, including brushing, mouthwash use, floss use, chewing gum and tongue cleaning aid in plaque control [50–55]. A reduction in plaque may increase the access of tastants to the taste buds on the tongue, increasing taste sensitivity due to reduction in bacterial load [56–59]. This is supported by the findings of a single-blind cross-over study (n = 16), which found that tongue cleaning improved taste sensation [60].

However, those having more sensitive taste perception may also be more inclined to practice good oral hygiene habits due to increased sensitivity to bacterial load. This hypothesis is supported by the pattern for bitter liking and oral hygiene habits presented here, with lower liking scores in those who performed oral hygiene habits more frequently. This is not suprising, as bitter is an aversive taste, and an inverse association between perception and liking has previously been reported. This finding supports the hypothesis that those who taste bitter more intensely may be more motivated that practice oral hygiene habits with increased frequency. This may be due to the more intense detection of the bitter metabolites produced by bacteria in the oral cavity. While bitter liking varied by frequency of a range of oral hygiene habits, sweet liking scores did not present clear patterns. This may be due to sweet being an appetitive rather than an aversive taste modality. Intersestingly, there was a U-shaped pattern in sweet liking and frequency of mouthwash use, with the highest mean sweet liking scores in those who use mouthwash less than weekly or never, and those who use it daily or more frequently, and the lowest scores in those who reported using mouthwash weekly. This variance may be due to the influence of mouthwash use on perception or may be related to preference or aversion to the taste of mouthwashes.

The patterns of variance of bitter and sweet perception (liking and intensity) were less clear for the self reported oral heath variables assessed. This may be due to the complex aetiology of oral pathologies, or due to lower accuracy of self-reporting of these variables. However, it was found here that mean bitter and sweet taste intensity scores, and mean bitter liking scores did vary by number of dental caries reported. These data support previous studies of the associations between dental caries and sweet and bitter taste perception that have focused on children [34, 61], and extend these findings into an adult sample. It may be hypothesized that these findings may reflect the consumption of a sugar-rich cariogenic diet leading to greater susceptibility to dental caries [23, 34, 62, 63]. However, associations here remained when adjustments were applied for diet quality scores. Additional, roles for taste receptors in the modulation of inflammation should be considered in future studies as this may be part of the complex aetiology of oral pathologies that has not yet been well-considered [27, 64].

This online exploratory survey allowed the recruitment of a large sample in an economical and efficient manner. Making these initial observations in this way is important to direct and justify future studies using genetic testing, chemical taste testing or interventions. However, due to the nature of the snowball recruitment design, participants were mostly female and highly educated, which may influence the outcomes and limited the generalisability of these data despite statistical adjustments for these factors being applied. However, this data serves to inform and justify future studies with a more targeted recruit for a more representable sample of the general population with a balanced distribution of gender, BMI, and education levels.

## Conclusions

In this exploratory study survey respondents who engaged in oral hygiene patterns most regularly had higher bitter and sweet intensity scores were higher, and bitter liking scores. However, conversely bitter intensity scores were also higher in those with more dental caries. Interestingly, although consumption of sugars is typically linked to oral health outcomes, sweet liking was not linked to frequency of engaging in oral hygiene habits, nor self-reported oral health outcomes. Although the cross-sectional and self-reported study design and adopted statistical strategy did not propose to assess causality, understanding the potential relationships between these factors is important to direct future studies with a more balance distribution of demographics, to identify populations at risk of oral disease. These finding together with future research are the necessary first step to identify future potential therapeutics, interventions or screening programs targeting taste perception or may contribute to the further investigations of taste related barriers or drivers of good oral hygiene habits.

## Supplementary Information

**Additional file 1.** Summary of potential assocations and adjusted models.

## Data Availability

Data can be accessed on request, with relevant ethics approvals, by contacting the corresponding author.

## Abbreviations

BMI: Body mass index
GLMS: General Labelled Magnitude Scales
PROP: 6-*n*-propylthiouracil (PROP)
