# Supplementary material for: Bitter and sweet taste perception: relationships to self-reported oral hygiene habits and oral health status in a survey of Australian adults
Source: BMC Oral Health. 2021 Oct 29;21:553. doi: 10.1186/s12903-021-01910-8 (PMC8555166; doi:10.1186/s12903-021-01910-8)
Supplement: Supplementary file 1 — Additional file 1. Summary of potential assocations and adjusted models. [file 12903_2021_1910_MOESM1_ESM.docx]

**Additional file 1 - Bitter and sweet taste perception correlates with self-reported oral hygiene habits & oral health outcomes in a survey of Australian adults.**

Kiranjit Kaur^1^, Dean Sculley^2^, Martin Veysey^3^, Mark Lucock^1^, Janet Wallace^4^, Emma L Beckett^1,5,6^

^1^ School of Environmental and Life Sciences, The University of Newcastle, NSW, Australia

^2^ School of Biomedical Sciences & Pharmacy, The University of Newcastle, NSW, Australia

^3^ School of Medicine & Public Health, The University of Newcastle, NSW, Australia

^4^ School of Health Sciences, The University of Newcastle, NSW, Australia

^5^ Hunter Medical Research Institute, NSW, Australia

^6^ Priority Research Centre for Physical Activity and Nutrition, The University of Newcastle, NSW, Australia


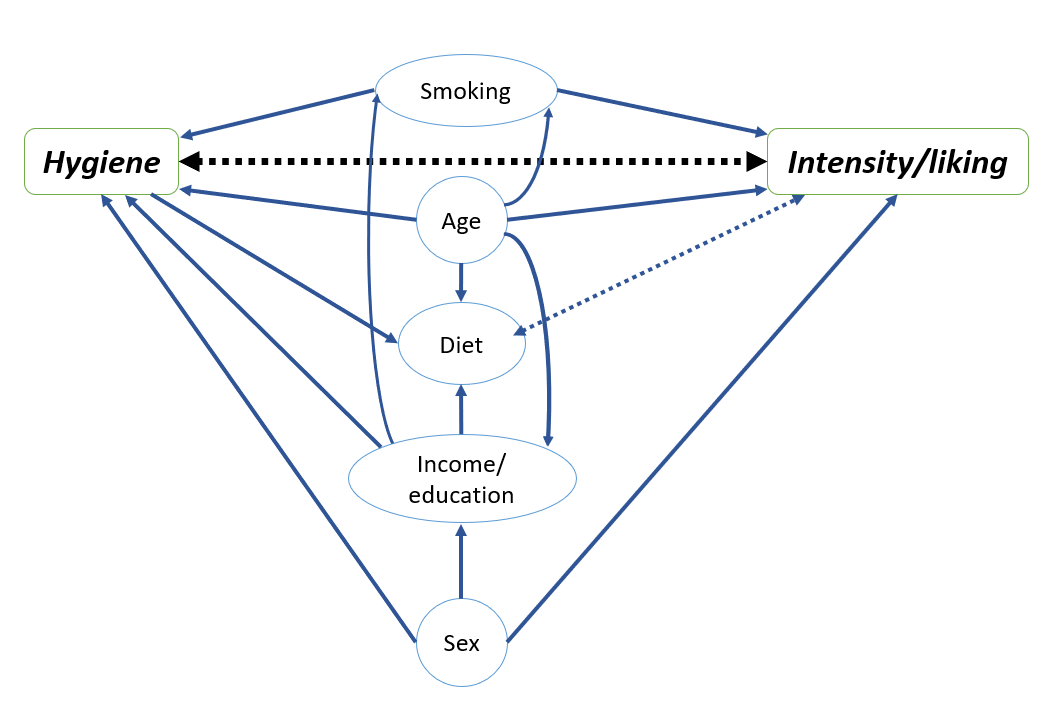


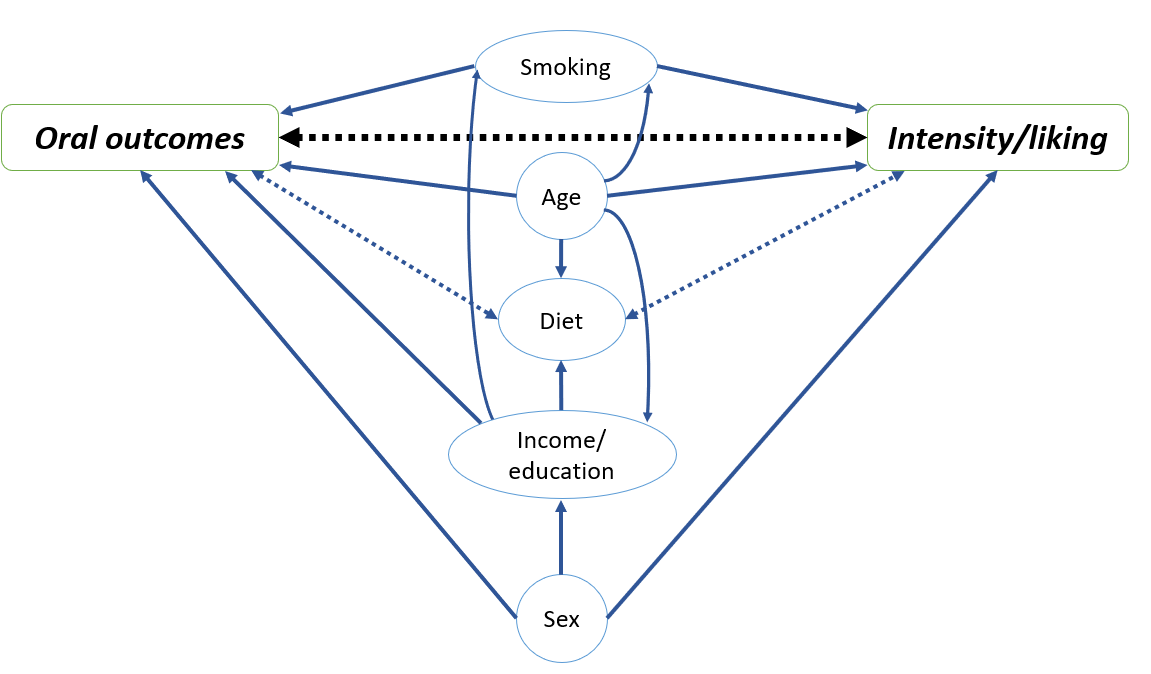


Supplementary figure 1. Potential associations between variables.

**Supplementary Table 1** – Least-squares means bitter intensity and oral hygiene habits adjusted for age, sex, and smoking status.

| **Oral Hygiene Habit** | **Level** | **Differences*** | | | **Least Sq Mean** |
| --- | --- | --- | --- | --- | --- |
| **Brushing frequency** | More than twice a day | A |  |  | 76.93178 |
|  | Twice a day |  | B |  | 50.38849 |
|  | Once a day |  |  | C | 43.91269 |
|  | Weekly |  | B | C | 43.51312 |
| **Mouthwash frequency** | Twice a day | A |  |  | 70.92955 |
|  | Less than weekly/never |  | B |  | 46.85049 |
|  | Weekly |  | B |  | 46.43282 |
|  | Once a day |  | B |  | 46.13121 |
| **Floss** | twice a day or more | A |  |  | 55.43372 |
|  | once a day | A | B |  | 51.75833 |
|  | weekly | A | B |  | 50.50757 |
|  | Less than weekly/Never |  | B |  | 45.70927 |
| **Chewing gum** | Twice a day | A |  |  | 75.51399 |
|  | Less than weekly/never |  | B |  | 46.69381 |
|  | Once a day |  | B |  | 44.48131 |
|  | Weekly |  | B |  | 42.96961 |
| **Tongue cleaning** | Twice a day | A |  |  | 59.57029 |
|  | Once a day |  | B |  | 49.06307 |
|  | Weekly |  | B |  | 46.33053 |
|  | Less than weekly/never |  | B |  | 45.56874 |

*Levels not linked by the same letter are significantly difference at P<0.05 (see supplementary table 2 for p-values, differences in 95% confidence intervals)

**Supplementary Table 2** – p-values, differences and 95% confidence intervals bitter intensity and oral hygiene habits adjusted for age, sex, and smoking status.

| Oral Hygiene Habit | **Level 1** | **Level 2** | **Difference** | **Lower CL** | **Upper CL** | **p-Value** |
| --- | --- | --- | --- | --- | --- | --- |
| **Brushing** | More than twice a day | Weekly | 33.4186 | 19.5836 | 47.25377 | <.0001 |
|  | More than twice a day | Once a day | 33.0191 | 24.1916 | 41.84659 | <.0001 |
|  | More than twice a day | Twice a day | 26.5433 | 18.0728 | 35.01379 | <.0001 |
|  | Twice a day | Weekly | 6.87537 | -4.9256 | 18.67635 | 0.4372 |
|  | Twice a day | Once a day | 6.4758 | 1.681 | 11.27058 | 0.003 |
|  | Once a day | Weekly | 0.39957 | -11.6713 | 12.47048 | 0.9998 |
| **Mouthwash** | Twice a day | Once a day | 24.79834 | 15.9394 | 33.65725 | <.0001 |
|  | Twice a day | Weekly | 24.49673 | 15.7185 | 33.27498 | <.0001 |
|  | Twice a day | Less than weekly/never | 24.07906 | 16.7508 | 31.40728 | <.0001 |
|  | Less than weekly/never | Once a day | 0.71929 | -5.874 | 7.3126 | 0.9922 |
|  | Less than weekly/never | Weekly | 0.41767 | -5.7406 | 6.57593 | 0.9981 |
|  | Weekly | Once a day | 0.30161 | -7.8505 | 8.45369 | 0.9997 |
| **Floss** | twice a day or more | Less than weekly/Never | 9.724454 | 0.82079 | 18.62812 | 0.026 |
|  | once a day | Less than weekly/Never | 6.049061 | -0.09337 | 12.19149 | 0.0554 |
|  | twice a day or more | weekly | 4.926151 | -4.31749 | 14.16979 | 0.5165 |
|  | weekly | Less than weekly/Never | 4.798303 | -0.7752 | 10.37181 | 0.1194 |
|  | twice a day or more | once a day | 3.675393 | -5.91416 | 13.26495 | 0.7564 |
|  | once a day | weekly | 1.250759 | -5.20885 | 7.71036 | 0.9593 |
| **Chewing Gum** | Twice a day | Weekly | 32.54438 | 22.3541 | 42.7347 | <.0001 |
|  | Twice a day | Once a day | 31.03268 | 21.0764 | 40.98901 | <.0001 |
|  | Twice a day | Less than weekly/never | 28.82018 | 20.8731 | 36.76728 | <.0001 |
|  | Less than weekly/never | Weekly | 3.72421 | -3.5927 | 11.04116 | 0.5557 |
|  | Less than weekly/never | Once a day | 2.21251 | -4.9159 | 9.3409 | 0.8544 |
|  | Once a day | Weekly | 1.5117 | -8.0751 | 11.09853 | 0.9773 |
| **Tongue cleaning** | Twice a day | Less than weekly/never | 14.00154 | 7.98699 | 20.0161 | <.0001 |
|  | Twice a day | Weekly | 13.23976 | 5.4475 | 21.03202 | <.0001 |
|  | Twice a day | Once a day | 10.50722 | 3.64717 | 17.36727 | 0.0005 |
|  | Once a day | Less than weekly/never | 3.49433 | -2.56173 | 9.55038 | 0.4459 |
|  | Once a day | Weekly | 2.73254 | -5.08906 | 10.55414 | 0.8046 |
|  | Weekly | Less than weekly/never | 0.76179 | -6.26423 | 7.78781 | 0.9924 |

**Supplementary Table 3**– Bitter intensity and oral hygiene habits adjusted for age, sex, smoking status, income, education and dietary index

| **Oral Hygiene Habit** | **Level** | **Differences*** | | | **Least Sq Mean** |
| --- | --- | --- | --- | --- | --- |
| **Brushing frequency** | More than twice a day | A |  |  | 84.77337 |
|  | Twice a day |  | B |  | 57.58289 |
|  | Once a day |  |  | C | 50.59693 |
|  | Weekly |  | B | C | 49.45507 |
| **Mouthwash frequency** | Twice a day | A |  |  | 80.41855 |
|  | Less than weekly/never |  | B |  | 55.66001 |
|  | Weekly |  | B |  | 55.49056 |
|  | Once a day |  | B |  | 55.06833 |
| **Floss** | twice a day and more than twice | A |  |  | 59.59299 |
|  | once a day | A |  |  | 56.07379 |
|  | weekly | A | B |  | 54.36451 |
|  | Less than weekly/Never |  | B |  | 49.09971 |
| **Chewing gum** | Twice a day | A |  |  | 88.23067 |
|  | Less than weekly/never |  | B |  | 57.65406 |
|  | Once a day |  | B |  | 55.28486 |
|  | Weekly |  | B |  | 54.19954 |
| **Tongue cleaning** | Twice a day | A |  |  | 59.5283 |
|  | Once a day |  | B |  | 49.17553 |
|  | Weekly |  | B |  | 45.80903 |
|  | Less than weekly/never |  | B |  | 45.33677 |

*Levels not linked by the same letter are significantly difference at P<0.05 (see supplementary table 4 for p-values, differences in 95% confidence intervals)

**Supplementary Table 4** – p-values, differences and 95% confidence intervals Bitter intensity and Oral hygiene habits outcomes adjusted for age, sex, smoking status, income, education and dietary index

| Oral Hygiene Habit | **Level 1** | **Level 2** | **Difference** | **Lower CL** | **Upper CL** | **p-Value** |
| --- | --- | --- | --- | --- | --- | --- |
| **Brushing** | More than twice a day | Weekly | 35.3183 | 21.191 | 49.44564 | <.0001 |
|  | More than twice a day | Once a day | 34.17644 | 25.0827 | 43.27015 | <.0001 |
|  | More than twice a day | Twice a day | 27.19048 | 18.4979 | 35.88311 | <.0001 |
|  | Twice a day | Weekly | 8.12782 | -3.9278 | 20.18338 | 0.3051 |
|  | Twice a day | Once a day | 6.98596 | 2.1234 | 11.84852 | 0.0013 |
|  | Once a day | Weekly | 1.14186 | -11.1001 | 13.38379 | 0.9951 |
| **Mouthwash** | Twice a day | Once a day | 25.35022 | 16.2849 | 34.41557 | <.0001 |
|  | Twice a day | Weekly | 24.92799 | 15.9429 | 33.91312 | <.0001 |
|  | Twice a day | Less than weekly/never | 24.75854 | 17.2326 | 32.28451 | <.0001 |
|  | Less than weekly/never | Once a day | 0.59167 | -6.0786 | 7.26195 | 0.9958 |
|  | Weekly | Once a day | 0.42223 | -7.8112 | 8.65563 | 0.9992 |
|  | Less than weekly/never | Weekly | 0.16944 | -6.05 | 6.3889 | 0.9999 |
| **Floss** | twice a day or more | Less than weekly/Never | 10.49328 | 1.44135 | 19.5452 | 0.0155 |
|  | once a day | Less than weekly/Never | 6.97408 | 0.62539 | 13.32277 | 0.0248 |
|  | weekly | Less than weekly/Never | 5.2648 | -0.36807 | 10.89767 | 0.0766 |
|  | twice a day or more | weekly | 5.22848 | -4.12228 | 14.57923 | 0.4741 |
|  | twice a day or more | once a day | 3.5192 | -6.19435 | 13.23274 | 0.7866 |
|  | once a day | weekly | 1.70928 | -4.89658 | 8.31514 | 0.9095 |
| **Chewing Gum** | Twice a day | Weekly | 34.03113 | 23.5334 | 44.52886 | <.0001 |
|  | Twice a day | Once a day | 32.94581 | 22.6868 | 43.20477 | <.0001 |
|  | Twice a day | Less than weekly/never | 30.57661 | 22.2277 | 38.92554 | <.0001 |
|  | Less than weekly/never | Weekly | 3.45452 | -3.9374 | 10.84643 | 0.6242 |
|  | Less than weekly/never | Once a day | 2.3692 | -4.828 | 9.56638 | 0.8311 |
|  | Once a day | Weekly | 1.08532 | -8.6037 | 10.77434 | 0.9916 |
| **Tongue cleaning** | Twice a day | Less than weekly/never | 14.19153 | 8.05712 | 20.32594 | <.0001 |
|  | Twice a day | Weekly | 13.71927 | 5.71681 | 21.72173 | <.0001 |
|  | Twice a day | Once a day | 10.35278 | 3.3503 | 17.35525 | 0.0009 |
|  | Once a day | Less than weekly/never | 3.83875 | -2.29844 | 9.97595 | 0.3725 |
|  | Once a day | Weekly | 3.36649 | -4.57544 | 11.30843 | 0.6942 |
|  | Twice a day | Less than weekly/never | 14.19153 | 8.05712 | 20.32594 | <.0001 |

**Supplementary Table 5** – Sweet intensity and Oral hygiene habits adjusted for age, sex, and smoking status.

| **Oral Hygiene Habit** | **Level** | **Differences*** | | | **Least Sq Mean** |
| --- | --- | --- | --- | --- | --- |
| **Brushing frequency** | More than twice a day | A |  |  | 85.88746 |
|  | Twice a day |  | B |  | 73.13435 |
|  | Once a day |  | B | C | 69.75534 |
|  | Weekly |  |  | C | 59.8830 |
| **Mouthwash frequency** | Twice a day | A |  |  | 82.85109 |
|  | Less than weekly/never |  | B |  | 71.79081 |
|  | Once a day |  | B |  | 70.97781 |
|  | Weekly |  | B |  | 68.66033 |
| **Floss** | twice a day and more than twice | A |  |  | 74.2632 |
|  | once a day | A |  |  | 74.12484 |
|  | weekly | A |  |  | 73.41917 |
|  | Less than weekly/Never | A |  |  | 69.75555 |
| **Chewing gum** | Twice a day | A |  |  | 86.5161 |
|  | Less than weekly/never |  | B |  | 71.25036 |
|  | Once a day |  | B |  | 68.64348 |
|  | Weekly |  | B |  | 67.00313 |
| **Tongue cleaning** | Twice a day | A |  |  | 75.74208 |
|  | Weekly | A |  |  | 72.33148 |
|  | Once a day | A |  |  | 71.55952 |
|  | Less than weekly/never | A |  |  | 71.06203 |

*Levels not linked by the same letter are significantly difference at P<0.05 (see supplementary table 6 for p-values)

**Supplementary Table 6** – p-values, differences and 95% confidence intervals sweet intensity and oral hygiene habits adjusted for age, sex, and smoking status.

| Oral Hygiene Habit | **Level 1** | **Level 2** | **Difference** | **Lower CL** | **Upper CL** | **p-Value** |
| --- | --- | --- | --- | --- | --- | --- |
| **Brushing** | More than twice a day | Weekly | 26.00442 | 14.5219 | 37.48697 | <.0001 |
|  | More than twice a day | Once a day | 16.13212 | 8.8057 | 23.45856 | <.0001 |
|  | Twice a day | Weekly | 13.25132 | 3.457 | 23.04563 | 0.003 |
|  | More than twice a day | Twice a day | 12.75311 | 5.723 | 19.78325 | <.0001 |
|  | Once a day | Weekly | 9.8723 | -0.146 | 19.89064 | 0.0551 |
|  | Twice a day | Once a day | 3.37901 | -0.6004 | 7.35848 | 0.1277 |
| **Mouthwash** | Twice a day | Weekly | 14.19076 | 6.90773 | 21.47379 | <.0001 |
|  | Twice a day | Once a day | 11.87329 | 4.52333 | 19.22324 | 0.0002 |
|  | Twice a day | Less than weekly/never | 11.06028 | 4.98029 | 17.14027 | <.0001 |
|  | Less than weekly/never | Weekly | 3.13048 | -1.97883 | 8.23979 | 0.3914 |
|  | Once a day | Weekly | 2.31747 | -4.44604 | 9.08099 | 0.8136 |
|  | Less than weekly/never | Once a day | 0.81301 | -4.65726 | 6.28327 | 0.9809 |
| **Floss** | twice a day or more | Less than weekly/Never | 4.507643 | -2.59871 | 11.61399 | 0.3599 |
|  | once a day | Less than weekly/Never | 4.36929 | -0.53321 | 9.27179 | 0.1 |
|  | weekly | Less than weekly/Never | 3.663613 | -0.78481 | 8.11203 | 0.1472 |
|  | twice a day or more | weekly | 0.84403 | -6.53366 | 8.22172 | 0.9911 |
|  | once a day | weekly | 0.705677 | -4.44998 | 5.86133 | 0.9849 |
|  | twice a day or more | once a day | 0.138353 | -7.51543 | 7.79213 | 1 |
| **Chewing Gum** | Twice a day | Weekly | 19.51296 | 11.0471 | 27.97881 | <.0001 |
|  | Twice a day | Once a day | 17.87261 | 9.6012 | 26.14407 | <.0001 |
|  | Twice a day | Less than weekly/never | 15.26574 | 8.6635 | 21.86799 | <.0001 |
|  | Less than weekly/never | Weekly | 4.24722 | -1.8315 | 10.32596 | 0.274 |
|  | Less than weekly/never | Once a day | 2.60687 | -3.3152 | 8.52896 | 0.6683 |
|  | Once a day | Weekly | 1.64035 | -6.3241 | 9.60484 | 0.9516 |
| **Tongue cleaning** | Twice a day | Less than weekly/never | 4.680042 | -0.24512 | 9.605208 | 0.0694 |
|  | Twice a day | Once a day | 4.18256 | -1.43496 | 9.800079 | 0.2212 |
|  | Twice a day | Weekly | 3.410594 | -2.97029 | 9.791474 | 0.5139 |
|  | Weekly | Less than weekly/never | 1.269448 | -4.48398 | 7.022874 | 0.9413 |
|  | Weekly | Once a day | 0.771966 | -5.63294 | 7.176875 | 0.9896 |
|  | Once a day | Less than weekly/never | 0.497482 | -4.46167 | 5.45663 | 0.9939 |

**Supplementary Table 7**– sweet intensity and Oral health outcomes adjusted for age, sex, smoking status, income, education and dietary index

| **Oral Hygiene Habit** | **Level** | **Differences*** | | | **Least Sq Mean** |
| --- | --- | --- | --- | --- | --- |
| **Brushing frequency** | More than twice a day | A |  |  | 95.93194 |
|  | Twice a day |  | B |  | 81.79454 |
|  | Once a day |  | B | C | 78.35575 |
|  | Weekly |  |  | C | 68.86233 |
| **Mouthwash frequency** | Twice a day | A |  |  | 92.48205 |
|  | Less than weekly/never |  | B |  | 80.95537 |
|  | Once a day |  | B |  | 80.24925 |
|  | Weekly |  | B |  | 78.03577 |
| **Floss** | once a day | A |  |  | 81.05615 |
|  | twice a day and more than twice | A |  |  | 80.93618 |
|  | weekly | A |  |  | 80.78219 |
|  | Less than weekly/Never | A |  |  | 77.1935 |
| **Chewing gum** | Twice a day | A |  |  | 98.71399 |
|  | Less than weekly/never |  | B |  | 82.39729 |
|  | Once a day |  | B |  | 80.08481 |
|  | Weekly |  | B |  | 78.46471 |
| **Tongue cleaning** | Twice a day | A |  |  | 82.16637 |
|  | Once a day | A | B |  | 78.18623 |
|  | Weekly | A | B |  | 77.74241 |
|  | Less than weekly/never |  | B |  | 77.00282 |

*Levels not linked by the same letter are significantly difference at P<0.05 (see supplementary table 8 for p-values, differences in 95% confidence intervals)

**Supplementary Table 8** – p-values, differences and 95% confidence intervals sweet intensity and Oral health outcomes adjusted for age, sex, smoking status, income, education and dietary index

| Oral Hygiene Habit | **Level 1** | **Level 2** | **Difference** | **Lower CL** | **Upper CL** | **p-Value** |
| --- | --- | --- | --- | --- | --- | --- |
| **Brushing** | More than twice a day | Weekly | 27.06961 | 15.4845 | 38.6547 | <.0001 |
|  | More than twice a day | Once a day | 17.57619 | 10.1189 | 25.03347 | <.0001 |
|  | More than twice a day | Twice a day | 14.1374 | 7.009 | 21.26577 | <.0001 |
|  | Twice a day | Weekly | 12.93221 | 3.0461 | 22.81835 | 0.0045 |
|  | Once a day | Weekly | 9.49342 | -0.5456 | 19.53239 | 0.0715 |
|  | Twice a day | Once a day | 3.43879 | -0.5487 | 7.42632 | 0.1184 |
| **Mouthwash** | Twice a day | Weekly | 14.44628 | 7.07111 | 21.82145 | <.0001 |
|  | Twice a day | Once a day | 12.2328 | 4.79178 | 19.67382 | 0.0002 |
|  | Twice a day | Less than weekly/never | 11.52668 | 5.34922 | 17.70414 | <.0001 |
|  | Less than weekly/never | Weekly | 2.9196 | -2.18545 | 8.02465 | 0.4539 |
|  | Once a day | Weekly | 2.21348 | -4.54465 | 8.97162 | 0.8332 |
|  | Less than weekly/never | Once a day | 0.70612 | -4.76897 | 6.18121 | 0.9873 |
| **Floss** | once a day | Less than weekly/Never | 3.862656 | -1.16988 | 8.89519 | 0.1975 |
|  | twice a day or more | Less than weekly/Never | 3.742686 | -3.43267 | 10.91805 | 0.535 |
|  | weekly | Less than weekly/Never | 3.588694 | -0.87642 | 8.05381 | 0.1638 |
|  | once a day | weekly | 0.273962 | -4.96243 | 5.51035 | 0.9991 |
|  | twice a day or more | weekly | 0.153993 | -7.25825 | 7.56623 | 0.9999 |
|  | once a day | twice a day or more | 0.119969 | -7.57985 | 7.81979 | 1 |
| **Chewing Gum** | Twice a day | Weekly | 20.24928 | 11.6092 | 28.88941 | <.0001 |
|  | Twice a day | Once a day | 18.62919 | 10.1856 | 27.0728 | <.0001 |
|  | Twice a day | Less than weekly/never | 16.31671 | 9.4451 | 23.18827 | <.0001 |
|  | Less than weekly/never | Weekly | 3.93257 | -2.1513 | 10.01647 | 0.3428 |
|  | Less than weekly/never | Once a day | 2.31248 | -3.6111 | 8.2361 | 0.7458 |
|  | Once a day | Weekly | 1.62009 | -6.3544 | 9.59461 | 0.9534 |
| **Tongue cleaning** | Twice a day | Less than weekly/never | 5.163547 | 0.19829 | 10.12881 | 0.0379 |
|  | Twice a day | Once a day | 4.423962 | -1.24392 | 10.09184 | 0.1849 |
|  | Twice a day | Weekly | 3.980142 | -2.49714 | 10.45742 | 0.3887 |
|  | Weekly | Less than weekly/never | 1.183406 | -4.55742 | 6.92423 | 0.9514 |
|  | Once a day | Less than weekly/never | 0.739585 | -4.22793 | 5.7071 | 0.9808 |
|  | Weekly | Once a day | 0.443821 | -5.98447 | 6.87211 | 0.998 |

**Supplementary Table 9**– Bitter intensity and Oral health outcomes adjusted for age, sex, and smoking status.

| **Oral Health Outcome** | **Level** | **Differences*** | | | **Least Sq Mean** |
| --- | --- | --- | --- | --- | --- |
| **Caries** | More than 3 | A |  |  | 53.67129 |
|  | 2 or 3 | A | B |  | 47.63099 |
|  | 1 |  | B | C | 44.54204 |
|  | None |  |  | C | 41.11942 |
| **Bleeding** | Yes | A |  |  | 50.58102 |
|  | Rarely/never | A |  |  | 48.05309 |
| **Missing teeth** | 1 | A |  |  | 54.50651 |
|  | 2 or 3 | A | B |  | 53.24561 |
|  | More than 3 | A | B |  | 48.30073 |
|  | None |  | B |  | 47.41458 |
| **Toothache** | Previously, but not in the last 12 months | A |  |  | 50.89888 |
|  | Sometimes | A |  |  | 49.84181 |
|  | Never | A |  |  | 47.45287 |
| **Dry Mouth** | I don't know | A |  |  | 58.60342 |
|  | Yes, but infrequently | A | B |  | 52.04158 |
|  | Yes, regularly |  | B |  | 47.23343 |
|  | No |  | B |  | 46.61143 |
| **Bad breath** | Don't answer | A |  |  | 56.35767 |
|  | Yes, but infrequently | A | B |  | 50.05101 |
|  | Yes, regularly | A | B |  | 48.49951 |
|  | No |  | B |  | 45.48921 |

*Levels not linked by the same letter are significantly difference at P<0.05 (see supplementary table 10 for p-values)

**Supplementary Table 10** – p-values, differences and 95% confidence intervals Bitter intensity and Oral health outcomes adjusted for age, sex, and smoking status.

| Oral Health Outcome | **Level 1** | **Level 2** | **Difference** | **Lower CL** | **Upper CL** | **p-Value** |
| --- | --- | --- | --- | --- | --- | --- |
| **Caries** | More than 3 | None | 12.55187 | 6.59563 | 18.50811 | <.0001 |
|  | More than 3 | 1 | 9.12925 | 1.70962 | 16.54887 | 0.0087 |
|  | 2 or 3 | None | 6.51157 | 0.12113 | 12.90202 | 0.0439 |
|  | More than 3 | 2 or 3 | 6.0403 | -0.08071 | 12.1613 | 0.0546 |
|  | 1 | None | 3.42263 | -4.14626 | 10.99151 | 0.6489 |
|  | 2 or 3 | 1 | 3.08895 | -4.71566 | 10.89356 | 0.7377 |
| **Bleeding** | Yes | Rarely/never | 2.527936 | -1.01743 | 6.073298 | 0.1619 |
| **Missing teeth** | 1 | None | 7.091924 | 0.14382 | 14.04002 | 0.0434 |
|  | 1 | More than 3 | 6.20578 | -5.21603 | 17.6276 | 0.4996 |
|  | 2 or 3 | None | 5.831029 | -3.25712 | 14.91918 | 0.3495 |
|  | 2 or 3 | More than 3 | 4.944885 | -7.67552 | 17.56528 | 0.7437 |
|  | 1 | 2 or 3 | 1.260895 | -9.17204 | 11.69383 | 0.9895 |
|  | More than 3 | None | 0.886144 | -9.18843 | 10.96072 | 0.9959 |
| **Toothache** | Previously, but not in the last 12 months | Never | 3.446007 | -1.52806 | 8.420077 | 0.2346 |
|  | Sometimes | Never | 2.38894 | -2.95212 | 7.729998 | 0.5448 |
|  | Previously, but not in the last 12 months | Sometimes | 1.057067 | -4.73894 | 6.853074 | 0.9037 |
| **Dry Mouth** | I don't know | No | 11.99199 | 2.79987 | 21.18411 | 0.0046 |
|  | I don't know | Yes, regularly | 11.36999 | 0.39395 | 22.34602 | 0.039 |
|  | I don't know | Yes, but infrequently | 6.56184 | -3.08443 | 16.20812 | 0.2973 |
|  | Yes, but infrequently | No | 5.43015 | -0.02239 | 10.88268 | 0.0514 |
|  | Yes, but infrequently | Yes, regularly | 4.80814 | -3.3442 | 12.96049 | 0.4261 |
|  | Yes, regularly | No | 0.622 | -6.87533 | 8.11934 | 0.9965 |
| **Bad breath** | Don't answer | No | 10.86846 | 1.21819 | 20.51873 | 0.0201 |
|  | Don't answer | Yes, regularly | 7.85816 | -3.75975 | 19.47607 | 0.3023 |
|  | Don't answer | Yes, but infrequently | 6.30666 | -2.8576 | 15.47092 | 0.2872 |
|  | Yes, but infrequently | No | 4.5618 | -0.72438 | 9.84798 | 0.118 |
|  | Yes, regularly | No | 3.01031 | -5.94685 | 11.96746 | 0.8223 |
|  | Yes, but infrequently | Yes, regularly | 1.5515 | -6.85622 | 9.95921 | 0.9644 |

**Supplementary Table 11**– Bitter intensity and Oral health outcomes adjusted for age, sex, smoking status, income, education and dietary index

| **Oral Health Outcome** | **Level** | **Differences*** | | | **Least Sq Mean** |
| --- | --- | --- | --- | --- | --- |
| **Caries** | More than 3 | A |  |  | 57.43852 |
|  | 2 or 3 | A | B |  | 51.51762 |
|  | 1 |  | B |  | 48.60783 |
|  | None |  | B |  | 45.07694 |
| **Bleeding** | Yes | A |  |  | 54.1613 |
|  | Rarely/never | A |  |  | 51.9607 |
| **Missing teeth** | 1 | A |  |  | 59.49822 |
|  | 2 or 3 | A | B |  | 58.16757 |
|  | None |  | B |  | 52.40382 |
|  | More than 3 | A | B |  | 51.844 |
| **Toothache** | Previously, but not in the last 12 months | A |  |  | 56.02733 |
|  | Sometimes | A |  |  | 54.35254 |
|  | Never | A |  |  | 52.65855 |
| **Dry Mouth** | I don't know | A |  |  | 62.49482 |
|  | Yes, but infrequently | A | B |  | 55.79774 |
|  | Yes, regularly |  | B |  | 51.0365 |
|  | No |  | B |  | 50.64279 |
| **Bad breath** | Don't answer | A |  |  | 63.43771 |
|  | Yes, but infrequently | A | B |  | 57.56447 |
|  | Yes, regularly | A | B |  | 55.88102 |
|  | No |  | B |  | 52.97443 |

*Levels not linked by the same letter are significantly difference at P<0.05 (see supplementary table 2 for p-values, differences in 95% confidence intervals)

**Supplementary Table 12** – p-values, differences and 95% confidence intervals Bitter intensity and Oral health outcomes adjusted for age, sex, smoking status, income, education and dietary index

| Oral Health Outcome | **Level 1** | **Level 2** | **Difference** | **Lower CL** | **Upper CL** | **p-Value** |
| --- | --- | --- | --- | --- | --- | --- |
| **Caries** | More than 3 | None | 12.36159 | 6.25083 | 18.47234 | <.0001 |
|  | More than 3 | 1 | 8.8307 | 1.28473 | 16.37667 | 0.0142 |
|  | 2 or 3 | None | 6.44068 | -0.04227 | 12.92363 | 0.0522 |
|  | More than 3 | 2 or 3 | 5.92091 | -0.36747 | 12.20928 | 0.0733 |
|  | 1 | None | 3.53089 | -4.14092 | 11.2027 | 0.6358 |
|  | 2 or 3 | 1 | 2.90979 | -4.98894 | 10.80852 | 0.778 |
| **Bleeding** | Yes | Rarely/never | 2.200604 | -1.41236 | 5.813568 | 0.232 |
| **Missing teeth** | 1 | More than 3 | 7.654223 | -4.00439 | 19.31283 | 0.3288 |
|  | 1 | None | 7.094398 | 0.09436 | 14.09444 | 0.0456 |
|  | 2 or 3 | More than 3 | 6.323566 | -6.51415 | 19.16128 | 0.5827 |
|  | 2 or 3 | None | 5.763742 | -3.47294 | 15.00043 | 0.3747 |
|  | 1 | 2 or 3 | 1.330656 | -9.23057 | 11.89188 | 0.9882 |
|  | None | More than 3 | 0.559824 | -9.96929 | 11.08894 | 0.9991 |
| **Toothache** | Previously, but not in the last 12 months | Never | 3.368772 | -1.64756 | 8.385106 | 0.2558 |
|  | Sometimes | Never | 1.693991 | -3.7951 | 7.183082 | 0.7485 |
|  | Previously, but not in the last 12 months | Sometimes | 1.674782 | -4.24233 | 7.59189 | 0.7837 |
| **Dry Mouth** | I don't know | No | 11.85203 | 2.46073 | 21.24333 | 0.0067 |
|  | I don't know | Yes, regularly | 11.45833 | 0.28732 | 22.62934 | 0.0419 |
|  | I don't know | Yes, but infrequently | 6.69708 | -3.0663 | 16.46046 | 0.29 |
|  | Yes, but infrequently | No | 5.15495 | -0.40794 | 10.71784 | 0.0805 |
|  | Yes, but infrequently | Yes, regularly | 4.76125 | -3.5152 | 13.03769 | 0.4486 |
|  | Yes, regularly | No | 0.3937 | -7.22126 | 8.00867 | 0.9992 |
| **Bad breath** | Don't answer | No | 10.46328 | 0.59626 | 20.33031 | 0.0328 |
|  | Don't answer | Yes, regularly | 7.55669 | -4.27767 | 19.39106 | 0.3538 |
|  | Don't answer | Yes, but infrequently | 5.87325 | -3.44029 | 15.18679 | 0.3651 |
|  | Yes, but infrequently | No | 4.59003 | -0.83287 | 10.01294 | 0.1297 |
|  | Yes, regularly | No | 2.90659 | -6.28327 | 12.09645 | 0.8473 |
|  | Yes, but infrequently | Yes, regularly | 1.68345 | -6.88095 | 10.24784 | 0.9575 |

**Supplementary Table 13**– sweet intensity and Oral health outcomes adjusted for age, sex, and smoking status.

| **Oral Health Outcome** | **Level** | **Differences*** | | | **Least Sq Mean** |
| --- | --- | --- | --- | --- | --- |
| **Caries** | More than 3 | A |  |  | 75.02478 |
|  | 2 or 3 | A |  |  | 71.57315 |
|  | 1 | A | B |  | 69.40388 |
|  | None |  | B |  | 65.93737 |
| **Bleeding** | Yes | A |  |  | 72.49706 |
|  | Rarely/never | A |  |  | 72.21092 |
| **Missing teeth** | 1 | A |  |  | 76.75461 |
|  | 2 or 3 | A |  |  | 73.81363 |
|  | None | A |  |  | 71.43572 |
|  | More than 3 | A |  |  | 68.34281 |
| **Toothache** | Previously, but not in the last 12 months | A |  |  | 73.61514 |
|  | Sometimes | A |  |  | 72.88075 |
|  | Never | A |  |  | 69.96697 |
| **Dry Mouth** | I don't know | A |  |  | 78.71874 |
|  | Yes, but infrequently | A | B |  | 73.74987 |
|  | No | A | B |  | 72.05849 |
|  | Yes, regularly |  | B |  | 68.36259 |
| **Bad breath** | Yes, regularly | A |  |  | 75.82757 |
|  | Yes, but infrequently | A |  |  | 74.21021 |
|  | No | A | B |  | 71.07704 |
|  | Don't answer |  | B |  | 65.72667 |

*Levels not linked by the same letter are significantly difference at P<0.05 (see supplementary table 14 for p-values)

**Supplementary Table 14** – p-values, differences and 95% confidence intervals sweet intensity and Oral health outcomes adjusted for age, sex, and smoking status.

| Oral Health Outcome | **Level 1** | **Level 2** | **Difference** | **Lower CL** | **Upper CL** | **p-Value** |
| --- | --- | --- | --- | --- | --- | --- |
| **Caries** | More than 3 | None | 9.08741 | 4.32816 | 13.84666 | <.0001 |
|  | 2 or 3 | None | 5.635775 | 0.52958 | 10.74197 | 0.0238 |
|  | More than 3 | 1 | 5.620907 | -0.30764 | 11.54946 | 0.0704 |
|  | 1 | None | 3.466503 | -2.58131 | 9.51432 | 0.4519 |
|  | More than 3 | 2 or 3 | 3.451635 | -1.43927 | 8.34254 | 0.2655 |
|  | 2 or 3 | 1 | 2.169272 | -4.06689 | 8.40543 | 0.8066 |
| **Bleeding** | Yes | Rarely/never | 0.286141 | -2.53607 | 3.10835 | 0.8422 |
| **Missing teeth** | 1 | More than 3 | 8.411807 | -0.68182 | 17.50543 | 0.0814 |
|  | 2 or 3 | More than 3 | 5.470825 | -4.57707 | 15.51872 | 0.4977 |
|  | 1 | None | 5.318896 | -0.21292 | 10.85072 | 0.0645 |
|  | None | More than 3 | 3.092911 | -4.9281 | 11.11392 | 0.753 |
|  | 1 | 2 or 3 | 2.940982 | -5.36534 | 11.2473 | 0.7981 |
|  | 2 or 3 | None | 2.377914 | -4.85774 | 9.61356 | 0.8319 |
| **Toothache** | Previously, but not in the last 12 months | Never | 3.648167 | -0.29391 | 7.590249 | 0.0764 |
|  | Sometimes | Never | 2.913778 | -1.31915 | 7.146707 | 0.2389 |
|  | Previously, but not in the last 12 months | Sometimes | 0.734389 | -3.8591 | 5.327878 | 0.9251 |
| **Dry Mouth** | I don't know | Yes, regularly | 10.35615 | 1.59183 | 19.12048 | 0.013 |
|  | I don't know | No | 6.66025 | -0.67963 | 14.00013 | 0.0907 |
|  | Yes, but infrequently | Yes, regularly | 5.38729 | -1.12233 | 11.89691 | 0.144 |
|  | I don't know | Yes, but infrequently | 4.96887 | -2.73365 | 12.67138 | 0.3446 |
|  | No | Yes, regularly | 3.6959 | -2.29069 | 9.6825 | 0.3845 |
|  | Yes, but infrequently | No | 1.69138 | -2.66245 | 6.04521 | 0.7487 |
| **Bad breath** | Yes, regularly | Don't answer | 10.10089 | 0.89277 | 19.30902 | 0.0251 |
|  | Yes, but infrequently | Don't answer | 8.48354 | 1.22013 | 15.74695 | 0.0145 |
|  | No | Don't answer | 5.35037 | -2.29824 | 12.99898 | 0.273 |
|  | Yes, regularly | No | 4.75053 | -2.34873 | 11.84979 | 0.3118 |
|  | Yes, but infrequently | No | 3.13317 | -1.05655 | 7.32289 | 0.2178 |
|  | Yes, regularly | Yes, but infrequently | 1.61736 | -5.04643 | 8.28114 | 0.9238 |

**Supplementary Table 15**– Sweet intensity and Oral health outcomes adjusted for age, sex, smoking status, income, education and dietary index

| **Oral Health Outcome** | **Level** | **Differences*** | | | **Least Sq Mean** |
| --- | --- | --- | --- | --- | --- |
| **Caries** | More than 3 | A |  |  | 82.74181 |
|  | 2 or 3 | A | B |  | 78.28213 |
|  | 1 |  | B | C | 76.47191 |
|  | None |  |  | C | 72.92244 |
| **Bleeding** | Rarely/never | A |  |  | 79.63176 |
|  | Yes | A |  |  | 79.57814 |
| **Missing teeth** | 1 | A |  |  | 84.30898 |
|  | 2 or 3 | A |  |  | 81.82907 |
|  | None | A |  |  | 79.30449 |
|  | More than 3 | A |  |  | 76.18837 |
| **Toothache** | Sometimes | A |  |  | 82.24065 |
|  | Previously, but not in the last 12 months | A |  |  | 82.20708 |
|  | Never | A |  |  | 78.50792 |
| **Dry Mouth** | I don't know | A |  |  | 86.59653 |
|  | Yes, but infrequently | A | B |  | 81.23109 |
|  | No |  | B |  | 78.91061 |
|  | Yes, regularly |  | B |  | 75.44064 |
| **Bad breath** | Yes, regularly | A |  |  | 85.59634 |
|  | Yes, but infrequently | A |  |  | 83.55169 |
|  | No | A | B |  | 80.07557 |
|  | Don't answer |  | B |  | 75.35856 |

*Levels not linked by the same letter are significantly difference at P<0.05 (see supplementary table 16 for p-values, differences in 95% confidence intervals)

**Supplementary Table 16** – p-values, differences and 95% confidence intervals sweet intensity and Oral health outcomes adjusted for age, sex, smoking status, income, education and dietary index

| Oral Health Outcome | **Level 1** | **Level 2** | **Difference** | **Lower CL** | **Upper CL** | **p-Value** |
| --- | --- | --- | --- | --- | --- | --- |
| **Caries** | More than 3 | None | 9.819372 | 5.0145 | 14.62424 | <.0001 |
|  | More than 3 | 1 | 6.269908 | 0.33653 | 12.20328 | 0.0337 |
|  | 2 or 3 | None | 5.359684 | 0.26216 | 10.45721 | 0.035 |
|  | More than 3 | 2 or 3 | 4.459688 | -0.48484 | 9.40422 | 0.0938 |
|  | 1 | None | 3.549464 | -2.48286 | 9.58179 | 0.4282 |
|  | 2 or 3 | 1 | 1.810219 | -4.40053 | 8.02097 | 0.8761 |
| **Bleeding** | Yes | Rarely/never | 0.053618 | -2.79117 | 2.898408 | 0.9705 |
| **Missing teeth** | 1 | More than 3 | 8.120611 | -1.05431 | 17.29553 | 0.1037 |
|  | 2 or 3 | More than 3 | 5.640698 | -4.46213 | 15.74353 | 0.4754 |
|  | 1 | None | 5.004492 | -0.50429 | 10.51328 | 0.0901 |
|  | None | More than 3 | 3.116119 | -5.16992 | 11.40216 | 0.7669 |
|  | 2 or 3 | None | 2.524578 | -4.74437 | 9.79353 | 0.8073 |
|  | 1 | 2 or 3 | 2.479913 | -5.8314 | 10.79123 | 0.8684 |
| **Toothache** | Sometimes | Never | 3.732727 | -0.56563 | 8.031088 | 0.1035 |
|  | Previously, but not in the last 12 months | Never | 3.699159 | -0.229 | 7.627316 | 0.0698 |
|  | Sometimes | Previously, but not in the last 12 months | 0.033568 | -4.59996 | 4.667098 | 0.9998 |
| **Dry Mouth** | I don't know | Yes, regularly | 11.15589 | 2.35817 | 19.95361 | 0.0063 |
|  | I don't know | No | 7.68592 | 0.28981 | 15.08203 | 0.0381 |
|  | Yes, but infrequently | Yes, regularly | 5.79045 | -0.72766 | 12.30856 | 0.1017 |
|  | I don't know | Yes, but infrequently | 5.36544 | -2.3237 | 13.05458 | 0.2751 |
|  | No | Yes, regularly | 3.46997 | -2.52719 | 9.46712 | 0.4434 |
|  | Yes, but infrequently | No | 2.32048 | -2.06057 | 6.70153 | 0.5218 |
| **Bad breath** | Yes, regularly | Don't answer | 10.23778 | 0.96893 | 19.50663 | 0.0237 |
|  | Yes, but infrequently | Don't answer | 8.19313 | 0.89863 | 15.48764 | 0.0206 |
|  | Yes, regularly | No | 5.52076 | -1.67687 | 12.7184 | 0.198 |
|  | No | Don't answer | 4.71701 | -3.01099 | 12.44502 | 0.3948 |
|  | Yes, but infrequently | No | 3.47612 | -0.77118 | 7.72342 | 0.1514 |
|  | Yes, regularly | Yes, but infrequently | 2.04464 | -4.66311 | 8.7524 | 0.8609 |

Supplementary Table 17 – Bitter liking and Oral hygiene habits adjusted for age, sex, and smoking status.

| **Oral Hygiene Habit** | **Level** | **Differences*** | | | **Least Sq Mean** |
| --- | --- | --- | --- | --- | --- |
| **Brushing frequency** | Twice a day | A |  |  | 66.17401 |
|  | Once a day |  | B |  | 61.33999 |
|  | Weekly | A | B |  | 56.94112 |
|  | More than twice a day |  | **B** |  | 54.13886 |
| **Mouthwash frequency** | Once a day | A |  |  | 68.74601 |
|  | Less than weekly/never | A | B |  | 63.7486 |
|  | Weekly | A | B |  | 62.82809 |
|  | Twice a day |  | B |  | 58.56603 |
| **Floss** | once a day | A |  |  | 65.5915 |
|  | weekly | A |  |  | 64.3051 |
|  | Less than weekly/Never | A |  |  | 63.89867 |
|  | twice a day and more than twice |  | B |  | 55.34475 |
| **Chewing gum** | Weekly | A |  |  | 65.44695 |
|  | Less than weekly/never | A |  |  | 64.92039 |
|  | Once a day | A |  |  | 64.52777 |
|  | Twice a day |  | B |  | 54.26626 |
| **Tongue cleaning** | Weekly | A |  |  | 65.28573 |
|  | Less than weekly/never | A |  |  | 64.24026 |
|  | Once a day | A |  |  | 64.04084 |
|  | Twice a day | A |  |  | 60.94845 |

*Levels not linked by the same letter are significantly difference at P<0.05 (see supplementary table 18 for p-values)

Supplementary Table 18 – p-values, differences and 95% confidence intervals bitter liking and oral hygiene habits adjusted for age, sex, and smoking status.

| Oral Hygiene Habit | **Level 1** | **Level 2** | **Difference** | **Lower CL** | **Upper CL** | **p-Value** |
| --- | --- | --- | --- | --- | --- | --- |
| **Brushing** | Twice a day | More than twice a day | 12.03516 | 3.7591 | 20.31121 | 0.0011 |
|  | Twice a day | Weekly | 9.23289 | -2.2972 | 20.76299 | 0.1664 |
|  | Once a day | More than twice a day | 7.20114 | -1.4237 | 15.82599 | 0.1383 |
|  | Twice a day | Once a day | 4.83402 | 0.1493 | 9.51874 | 0.0401 |
|  | Once a day | Weekly | 4.39887 | -7.3949 | 16.19269 | 0.7714 |
|  | Weekly | More than twice a day | 2.80227 | -10.7153 | 16.31978 | 0.9507 |
| **Mouthwash** | Once a day | Twice a day | 10.17998 | 1.57352 | 18.78644 | 0.0129 |
|  | Once a day | Weekly | 5.91792 | -2.00184 | 13.83769 | 0.2184 |
|  | Less than weekly/never | Twice a day | 5.18258 | -1.93682 | 12.30197 | 0.2395 |
|  | Once a day | Less than weekly/never | 4.99741 | -1.40802 | 11.40284 | 0.1852 |
|  | Weekly | Twice a day | 4.26206 | -4.26604 | 12.79016 | 0.5709 |
|  | Less than weekly/never | Weekly | 0.92052 | -5.06225 | 6.90328 | 0.9788 |
| **Floss** | once a day | twice a day or more | 10.24676 | 1.45724 | 19.03627 | 0.0148 |
|  | weekly | twice a day or more | 8.96036 | 0.4879 | 17.43282 | 0.0335 |
|  | Less than weekly/Never | twice a day or more | 8.55392 | 0.39308 | 16.71477 | 0.0358 |
|  | once a day | Less than weekly/Never | 1.69283 | -3.93715 | 7.32281 | 0.8657 |
|  | once a day | weekly | 1.2864 | -4.6343 | 7.20709 | 0.9438 |
|  | weekly | Less than weekly/Never | 0.40643 | -4.70208 | 5.51495 | 0.9969 |
| **Chewing Gum** | Weekly | Twice a day | 11.18069 | 1.15182 | 21.20956 | 0.0219 |
|  | Less than weekly/never | Twice a day | 10.65413 | 2.83293 | 18.47533 | 0.0027 |
|  | Once a day | Twice a day | 10.26151 | 0.46291 | 20.0601 | 0.036 |
|  | Weekly | Once a day | 0.91918 | -8.51576 | 10.35413 | 0.9944 |
|  | Weekly | Less than weekly/never | 0.52656 | -6.67447 | 7.7276 | 0.9976 |
|  | Less than weekly/never | Once a day | 0.39262 | -6.62284 | 7.40808 | 0.9989 |
| **Tongue cleaning** | Weekly | Twice a day | 4.337275 | -3.026 | 11.70055 | 0.4272 |
|  | Less than weekly/never | Twice a day | 3.29181 | -2.39163 | 8.97525 | 0.4425 |
|  | Once a day | Twice a day | 3.092382 | -3.39 | 9.57477 | 0.6083 |
|  | Weekly | Once a day | 1.244892 | -6.14611 | 8.63589 | 0.9726 |
|  | Weekly | Less than weekly/never | 1.045464 | -5.59375 | 7.68468 | 0.9774 |
|  | Less than weekly/never | Once a day | 0.199428 | -5.52323 | 5.92208 | 0.9997 |

**Supplementary Table 19**– Bitter liking and Oral health outcomes adjusted for age, sex, smoking status, income, education and dietary index

| **Oral Hygiene Habit** | **Level** | **Differences*** | | | **Least Sq Mean** |
| --- | --- | --- | --- | --- | --- |
| **Brushing frequency** | Twice a day | A |  |  | 71.22496 |
|  | Once a day | A | B |  | 67.04377 |
|  | Weekly | A | B |  | 62.79547 |
|  | More than twice a day |  | B |  | 59.84985 |
| **Mouthwash frequency** | Once a day | A |  |  | 73.31365 |
|  | Less than weekly/never | A | B |  | 68.96772 |
|  | Weekly | A | B |  | 67.60234 |
|  | Twice a day |  | B |  | 64.25609 |
| **Floss** | Less than weekly/Never | A |  |  | 69.74489 |
|  | weekly | A |  |  | 69.55543 |
|  | once a day | A |  |  | 69.19065 |
|  | twice a day and more than twice |  | B |  | 59.58813 |
| **Chewing gum** | Weekly | A |  |  | 68.82767 |
|  | Less than weekly/never | A |  |  | 68.45986 |
|  | Once a day | A | B |  | 68.33754 |
|  | Twice a day |  | B |  | 58.59024 |
| **Tongue cleaning** | Weekly | A |  |  | 71.4693 |
|  | Less than weekly/never | A |  |  | 70.71403 |
|  | Once a day | A |  |  | 69.90292 |
|  | Twice a day | A |  |  | 68.56465 |

*Levels not linked by the same letter are significantly difference at P<0.05 (see supplementary table 20 for p-values, differences in 95% confidence intervals)

**Supplementary Table 20** – p-values, differences and 95% confidence intervals bitter liking and Oral health outcomes adjusted for age, sex, smoking status, income, education and dietary index

| Oral Hygiene Habit | **Level 1** | **Level 2** | **Difference** | **Lower CL** | **Upper CL** | **p-Value** |
| --- | --- | --- | --- | --- | --- | --- |
| **Brushing** | More than twice a day | Weekly | 4.87081 | -8.61171 | 18.35333 | 0.7881 |
|  | Once a day | Weekly | 4.357785 | -7.32538 | 16.04095 | 0.7714 |
|  | More than twice a day | Twice a day | 2.652648 | -5.64321 | 10.94851 | 0.843 |
|  | Twice a day | Weekly | 2.218161 | -9.28715 | 13.72347 | 0.9597 |
|  | Once a day | Twice a day | 2.139624 | -2.50099 | 6.78024 | 0.6344 |
|  | More than twice a day | Once a day | 0.513025 | -8.16561 | 9.19166 | 0.9987 |
| **Mouthwash** | Once a day | Twice a day | 9.057559 | 0.42385 | 17.69127 | 0.0355 |
|  | Once a day | Weekly | 5.711309 | -2.13006 | 13.55268 | 0.239 |
|  | Less than weekly/never | Twice a day | 4.711623 | -2.45599 | 11.87924 | 0.3276 |
|  | Once a day | Less than weekly/never | 4.345936 | -2.00673 | 10.6986 | 0.2923 |
|  | Weekly | Twice a day | 3.34625 | -5.21105 | 11.90355 | 0.7448 |
|  | Less than weekly/never | Weekly | 1.365373 | -4.55794 | 7.28869 | 0.9338 |
| **Floss** | Less than weekly/Never | twice a day or more | 10.15676 | 2.03748 | 18.27604 | 0.0073 |
|  | weekly | twice a day or more | 9.96729 | 1.57997 | 18.35462 | 0.0123 |
|  | once a day | twice a day or more | 9.60252 | 0.88978 | 18.31525 | 0.0241 |
|  | Less than weekly/Never | once a day | 0.55424 | -5.14033 | 6.24881 | 0.9945 |
|  | weekly | once a day | 0.36478 | -5.56046 | 6.29002 | 0.9986 |
|  | Less than weekly/Never | weekly | 0.18947 | -4.86304 | 5.24197 | 0.9997 |
| **Chewing Gum** | Weekly | Twice a day | 10.23743 | 0.07993 | 20.39492 | 0.0474 |
|  | Less than weekly/never | Twice a day | 9.86962 | 1.79128 | 17.94796 | 0.0094 |
|  | Once a day | Twice a day | 9.7473 | -0.17916 | 19.67376 | 0.0564 |
|  | Weekly | Once a day | 0.49013 | -8.88487 | 9.86512 | 0.9991 |
|  | Weekly | Less than weekly/never | 0.36781 | -6.78453 | 7.52014 | 0.9992 |
|  | Less than weekly/never | Once a day | 0.12232 | -6.84159 | 7.08623 | 1 |
| **Tongue cleaning** | Weekly | Twice a day | 2.904654 | -4.50448 | 10.31379 | 0.7433 |
|  | Less than weekly/never | Twice a day | 2.149381 | -3.53021 | 7.82897 | 0.7635 |
|  | Weekly | Once a day | 1.566386 | -5.78671 | 8.91948 | 0.9468 |
|  | Once a day | Twice a day | 1.338268 | -5.14503 | 7.82156 | 0.9512 |
|  | Less than weekly/never | Once a day | 0.811113 | -4.87105 | 6.49328 | 0.983 |
|  | Weekly | Less than weekly/never | 0.755273 | -5.81146 | 7.32201 | 0.9909 |

Supplementary Table 21 – Sweet liking and Oral hygiene habits adjusted for age, sex, and smoking status.

| **Oral Hygiene Habit** | **Level** | **Differences*** | | | **Least Sq Mean** |
| --- | --- | --- | --- | --- | --- |
| **Brushing frequency** | Once a day | A |  |  | 66.89895 |
|  | Weekly | A |  |  | 65.22478 |
|  | More than twice a day | A |  |  | 64.9873 |
|  | Twice a day | A |  |  | 63.7254 |
| **Mouthwash frequency** | Less than weekly/never | A |  |  | 66.65561 |
|  | Twice a day | A | B |  | 66.59481 |
|  | Once a day | A | B |  | 65.8121 |
|  | Weekly |  | B |  | 58.69494 |
| **Floss** | Less than weekly/Never | A |  |  | 66.95156 |
|  | weekly | A | B |  | 66.19554 |
|  | once a day | A | B |  | 63.14502 |
|  | twice a day and more than twice |  | B |  | 58.30603 |
| **Chewing gum** | Less than weekly/never | A |  |  | 65.80249 |
|  | Once a day | A |  |  | 65.66422 |
|  | Twice a day | A |  |  | 62.98416 |
|  | Weekly | A |  |  | 60.99453 |
| **Tongue cleaning** | Less than weekly/never | A |  |  | 66.32365 |
|  | Twice a day | A |  |  | 65.13987 |
|  | Weekly | A |  |  | 64.32289 |
|  | Once a day | A |  |  | 62.73127 |

*Levels not linked by the same letter are significantly difference at P<0.05 (see supplementary table 22 for p-values)

Supplementary Table 22 – p-values, differences and 95% confidence intervals sweet liking and oral hygiene habits adjusted for age, sex, and smoking status.

| Oral Hygiene Habit | **Level 1** | **Level 2** | **Difference** | **Lower CL** | **Upper CL** | **p-Value** |
| --- | --- | --- | --- | --- | --- | --- |
| **Brushing** | Once a day | Twice a day | 3.173485 | -1.5837 | 7.9307 | 0.3146 |
|  | Once a day | More than twice a day | 1.911653 | -6.8467 | 10.66999 | 0.9431 |
|  | Once a day | Weekly | 1.674168 | -10.3022 | 13.65051 | 0.984 |
|  | Weekly | Twice a day | 1.499317 | -10.2092 | 13.20785 | 0.9876 |
|  | More than twice a day | Twice a day | 1.261832 | -7.1423 | 9.66596 | 0.9803 |
|  | Weekly | More than twice a day | 0.237485 | -13.4892 | 13.9642 | 1 |
| **Mouthwash** | Less than weekly/never | Weekly | 7.960672 | 1.99799 | 13.92335 | 0.0035 |
|  | Twice a day | Weekly | 7.89987 | -0.59959 | 16.39933 | 0.0792 |
|  | Once a day | Weekly | 7.117161 | -0.77602 | 15.01034 | 0.0939 |
|  | Less than weekly/never | Once a day | 0.843511 | -5.54041 | 7.22743 | 0.9864 |
|  | Twice a day | Once a day | 0.782709 | -7.79485 | 9.36027 | 0.9954 |
|  | Less than weekly/never | Twice a day | 0.060802 | -7.03469 | 7.15629 | 1 |
| **Floss** | Less than weekly/Never | twice a day or more | 8.645527 | 0.4909 | 16.80016 | 0.0328 |
|  | weekly | twice a day or more | 7.88951 | -0.57649 | 16.35551 | 0.078 |
|  | once a day | twice a day or more | 4.838987 | -3.94383 | 13.6218 | 0.4873 |
|  | Less than weekly/Never | once a day | 3.80654 | -1.81915 | 9.43223 | 0.302 |
|  | weekly | once a day | 3.050523 | -2.86566 | 8.9667 | 0.5448 |
|  | Less than weekly/Never | weekly | 0.756018 | -4.3486 | 5.86064 | 0.9811 |
| **Chewing Gum** | Less than weekly/never | Weekly | 4.807962 | -2.45088 | 12.0668 | 0.3209 |
|  | Once a day | Weekly | 4.669695 | -4.84098 | 14.18038 | 0.5853 |
|  | Less than weekly/never | Twice a day | 2.818333 | -5.06565 | 10.70232 | 0.7934 |
|  | Once a day | Twice a day | 2.680067 | -7.19718 | 12.55732 | 0.8973 |
|  | Twice a day | Weekly | 1.989629 | -8.11975 | 12.099 | 0.9573 |
|  | Less than weekly/never | Once a day | 0.138267 | -6.9335 | 7.21004 | 1 |
| **Tongue cleaning** | Less than weekly/never | Once a day | 3.592377 | -2.12715 | 9.311902 | 0.3688 |
|  | Twice a day | Once a day | 2.4086 | -4.07024 | 8.887444 | 0.7732 |
|  | Less than weekly/never | Weekly | 2.000753 | -4.63484 | 8.636342 | 0.8648 |
|  | Weekly | Once a day | 1.591624 | -5.79534 | 8.978586 | 0.9451 |
|  | Less than weekly/never | Twice a day | 1.183777 | -4.49656 | 6.864109 | 0.9499 |
|  | Twice a day | Weekly | 0.816977 | -6.54227 | 8.176225 | 0.9918 |

**Supplementary Table 23**– sweet liking and Oral hygiene habits adjusted for age, sex, smoking status, income, education and dietary index

| **Oral Hygiene Habit** | **Level** | **Differences*** | | | **Least Sq Mean** |
| --- | --- | --- | --- | --- | --- |
| **Brushing frequency** | More than twice a day | A |  |  | 94.79946 |
|  | Once a day | A |  |  | 94.28643 |
|  | Twice a day | A |  |  | 92.14681 |
|  | Weekly | A |  |  | 89.92865 |
| **Mouthwash frequency** | Twice a day | A |  |  | 95.61438 |
|  | Once a day | A |  |  | 94.3623 |
|  | Less than weekly/never | A |  |  | 94.02452 |
|  | Weekly |  | B |  | 86.12332 |
| **Floss** | Less than weekly/Never | A |  |  | 95.0156 |
|  | weekly | A |  |  | 94.33958 |
|  | once a day | A |  |  | 91.70436 |
|  | twice a day and more than twice | A |  |  | 88.55294 |
| **Chewing gum** | Less than weekly/never | A |  |  | 93.04543 |
|  | Once a day | A |  |  | 92.83443 |
|  | Twice a day | A |  |  | 92.73653 |
|  | Weekly | A |  |  | 88.23136 |
| **Tongue cleaning** | Less than weekly/never | A |  |  | 94.8209 |
|  | Twice a day | A |  |  | 94.00288 |
|  | Weekly | A |  |  | 92.25614 |
|  | Once a day | A |  |  | 91.8739 |

*Levels not linked by the same letter are significantly difference at P<0.05 (see supplementary table 24 for p-values, differences in 95% confidence intervals)

**Supplementary Table 24** – p-values, differences and 95% confidence intervals sweet liking and Oral hygiene habits outcomes adjusted for age, sex, smoking status, income, education and dietary index

| Oral Hygiene Habit | **Level 1** | **Level 2** | **Difference** | **Lower CL** | **Upper CL** | **p-Value** |
| --- | --- | --- | --- | --- | --- | --- |
| **Brushing** | More than twice a day | Weekly | 4.87081 | -8.61171 | 18.35333 | 0.7881 |
|  | Once a day | Weekly | 4.357785 | -7.32538 | 16.04095 | 0.7714 |
|  | More than twice a day | Twice a day | 2.652648 | -5.64321 | 10.94851 | 0.843 |
|  | Twice a day | Weekly | 2.218161 | -9.28715 | 13.72347 | 0.9597 |
|  | Once a day | Twice a day | 2.139624 | -2.50099 | 6.78024 | 0.6344 |
|  | More than twice a day | Once a day | 0.513025 | -8.16561 | 9.19166 | 0.9987 |
| **Mouthwash** | Twice a day | Weekly | 9.491067 | 1.16245 | 17.81968 | 0.0181 |
|  | Once a day | Weekly | 8.238979 | 0.60716 | 15.87079 | 0.0285 |
|  | Less than weekly/never | Weekly | 7.9012 | 2.13618 | 13.66622 | 0.0025 |
|  | Twice a day | Less than weekly/never | 1.589867 | -5.3862 | 8.56594 | 0.9359 |
|  | Twice a day | Once a day | 1.252088 | -7.15089 | 9.65507 | 0.9807 |
|  | Once a day | Less than weekly/never | 0.337779 | -5.84512 | 6.52068 | 0.999 |
| **Floss** | Less than weekly/Never | twice a day or more | 6.462668 | -1.52998 | 14.45532 | 0.1596 |
|  | weekly | twice a day or more | 5.78664 | -2.46987 | 14.04315 | 0.2714 |
|  | Less than weekly/Never | once a day | 3.311245 | -2.29451 | 8.917 | 0.4247 |
|  | once a day | twice a day or more | 3.151423 | -5.42542 | 11.72827 | 0.7794 |
|  | weekly | once a day | 2.635217 | -3.19761 | 8.46804 | 0.6495 |
|  | Less than weekly/Never | weekly | 0.676028 | -4.29767 | 5.64973 | 0.9852 |
| **Chewing Gum** | Less than weekly/never | Weekly | 4.814068 | -2.24008 | 11.86821 | 0.2944 |
|  | Once a day | Weekly | 4.603067 | -4.64322 | 13.84935 | 0.574 |
|  | Twice a day | Weekly | 4.50517 | -5.51287 | 14.52321 | 0.6529 |
|  | Less than weekly/never | Twice a day | 0.308899 | -7.65853 | 8.27633 | 0.9996 |
|  | Less than weekly/never | Once a day | 0.211001 | -6.65731 | 7.07931 | 0.9998 |
|  | Once a day | Twice a day | 0.097898 | -9.69229 | 9.88808 | 1 |
| **Tongue cleaning** | Less than weekly/never | Once a day | 2.947001 | -2.612 | 8.506001 | 0.521 |
|  | Less than weekly/never | Weekly | 2.564761 | -3.85963 | 8.989156 | 0.7325 |
|  | Twice a day | Once a day | 2.128985 | -4.21378 | 8.471749 | 0.8228 |
|  | Twice a day | Weekly | 1.746745 | -5.50179 | 8.99528 | 0.9253 |
|  | Less than weekly/never | Twice a day | 0.818016 | -4.73846 | 6.374495 | 0.9814 |
|  | Weekly | Once a day | 0.38224 | -6.81147 | 7.575955 | 0.9991 |

**Supplementary Table 25**– Bitter liking and Oral health outcomes adjusted for age, sex, and smoking status.

| **Oral Health Outcome** | **Level** | **Differences*** | | | **Least Sq Mean** |
| --- | --- | --- | --- | --- | --- |
| **Caries** | 1 | A |  |  | 67.33969 |
|  | 2 or 3 | A |  |  | 65.18554 |
|  | More than 3 | A |  |  | 63.14551 |
|  | None | A |  |  | 60.42187 |
| **Bleeding** | Rarely/never | A |  |  | 64.20058 |
|  | Yes | A |  |  | 63.25388 |
| **Missing teeth** | None | A |  |  | 65.50313 |
|  | 2 or 3 | A |  |  | 62.61598 |
|  | More than 3 | A |  |  | 61.02084 |
|  | 1 | A |  |  | 59.91044 |
| **Toothache** | Sometimes | A |  |  | 64.64757 |
|  | Never | A |  |  | 64.26713 |
|  | Previously, but not in the last 12 months | A |  |  | 62.32375 |
| **Dry Mouth** | Yes, regularly | A |  |  | 66.1448 |
|  | No | A |  |  | 65.29934 |
|  | Yes, but infrequently | A | B |  | 62.90742 |
|  | I don't know |  | B |  | 54.64965 |
| **Bad breath** | Yes, regularly | A |  |  | 65.84865 |
|  | No | A |  |  | 64.20554 |
|  | Yes, but infrequently | A |  |  | 63.84079 |
|  | Don't answer | A |  |  | 60.51418 |

*Levels not linked by the same letter are significantly difference at P<0.05 (see supplementary table 26 for p-values)

**Supplementary Table 26** – p-values, differences and 95% confidence intervals Bitter liking and Oral health outcomes adjusted for age, sex, and smoking status.

| Oral Health Outcome | **Level 1** | **Level 2** | **Difference** | **Lower CL** | **Upper CL** | **p-Value** |
| --- | --- | --- | --- | --- | --- | --- |
| **Caries** | 1 | None | 6.917823 | -0.15246 | 13.98811 | 0.0578 |
|  | 2 or 3 | None | 4.763673 | -1.2058 | 10.73314 | 0.1688 |
|  | 1 | More than 3 | 4.194178 | -2.73668 | 11.12503 | 0.4027 |
|  | More than 3 | None | 2.723646 | -2.84022 | 8.28752 | 0.5878 |
|  | 1 | 2 or 3 | 2.154151 | -5.13632 | 9.44462 | 0.8717 |
|  | 2 or 3 | More than 3 | 2.040027 | -3.67775 | 7.7578 | 0.7944 |
| **Bleeding** | Yes | Rarely/never | 0.946701 | -2.29858 | 4.191983 | 0.5668 |
| **Missing teeth** | None | 1 | 5.592688 | -0.7815 | 11.96688 | 0.1085 |
|  | None | More than 3 | 4.48229 | -4.76013 | 13.72472 | 0.5952 |
|  | None | 2 or 3 | 2.887145 | -5.45033 | 11.22462 | 0.8087 |
|  | 2 or 3 | 1 | 2.705543 | -6.86564 | 12.27673 | 0.8856 |
|  | 2 or 3 | More than 3 | 1.595146 | -9.98282 | 13.17311 | 0.9846 |
|  | More than 3 | 1 | 1.110398 | -9.36798 | 11.58878 | 0.9929 |
| **Toothache** | Sometimes | Previously, but not in the last 12 months | 2.323816 | -2.98104 | 7.628668 | 0.5585 |
|  | Never | Previously, but not in the last 12 months | 1.943379 | -2.60919 | 6.495945 | 0.575 |
|  | Sometimes | Never | 0.380437 | -4.50802 | 5.268892 | 0.9817 |
| **Dry Mouth** | Yes, regularly | I don't know | 11.49515 | 1.42951 | 21.56079 | 0.0178 |
|  | No | I don't know | 10.64969 | 2.22 | 19.07938 | 0.0066 |
|  | Yes, but infrequently | I don't know | 8.25777 | -0.58841 | 17.10394 | 0.0772 |
|  | Yes, regularly | Yes, but infrequently | 3.23739 | -4.23877 | 10.71355 | 0.6797 |
|  | No | Yes, but infrequently | 2.39192 | -2.60836 | 7.3922 | 0.6061 |
|  | Yes, regularly | No | 0.84547 | -6.03001 | 7.72094 | 0.989 |
| **Bad breath** | Yes, regularly | Don't answer | 5.334473 | -5.36903 | 16.03798 | 0.5732 |
|  | No | Don't answer | 3.691357 | -5.19937 | 12.58208 | 0.7079 |
|  | Yes, but infrequently | Don't answer | 3.326608 | -5.11636 | 11.76958 | 0.7404 |
|  | Yes, regularly | Yes, but infrequently | 2.007865 | -5.7381 | 9.75383 | 0.9091 |
|  | Yes, regularly | No | 1.643116 | -6.60905 | 9.89528 | 0.9559 |
|  | No | Yes, but infrequently | 0.364749 | -4.50537 | 5.23487 | 0.9974 |

**Supplementary Table 27**– bitter liking and Oral health outcomes adjusted for age, sex, smoking status, income, education and dietary index

| **Oral Health Outcome** | **Level** | **Differences*** | | | **Least Sq Mean** |
| --- | --- | --- | --- | --- | --- |
| **Caries** | 1 | A |  |  | 72.89799 |
|  | 2 or 3 | A | B |  | 70.33836 |
|  | More than 3 | A | B |  | 69.95067 |
|  | None |  | B |  | 65.49764 |
| **Bleeding** | Rarely/never | A |  |  | 69.89468 |
|  | Yes | A |  |  | 69.08359 |
| **Missing teeth** | None | A |  |  | 69.98476 |
|  | More than 3 | A |  |  | 69.383 |
|  | 2 or 3 | A |  |  | 68.86715 |
|  | 1 | A |  |  | 64.90237 |
| **Toothache** | Sometimes | A |  |  | 71.26822 |
|  | Never | A |  |  | 69.24888 |
|  | Previously, but not in the last 12 months | A |  |  | 67.76248 |
| **Dry Mouth** | Yes, regularly | A | B |  | 71.42543 |
|  | No | A |  |  | 69.99678 |
|  | Yes, but infrequently | A | B |  | 68.82513 |
|  | I don't know |  | B |  | 61.39884 |
| **Bad breath** | Yes, regularly | A |  |  | 72.1937 |
|  | Yes, but infrequently | A |  |  | 69.662 |
|  | No | A |  |  | 69.5113 |
|  | Don't answer | A |  |  | 67.90036 |

*Levels not linked by the same letter are significantly difference at P<0.05 (see supplementary table 28 for p-values, differences in 95% confidence intervals)

**Supplementary Table 28** – p-values, differences and 95% confidence intervals nitter liking and Oral health outcomes adjusted for age, sex, smoking status, income, education and dietary index

| Oral Health Outcome | **Level 1** | **Level 2** | **Difference** | **Lower CL** | **Upper CL** | **p-Value** |
| --- | --- | --- | --- | --- | --- | --- |
| **Caries** | 1 | None | 7.400352 | 0.41424 | 14.38646 | 0.033 |
|  | 2 or 3 | None | 4.840721 | -1.06279 | 10.74423 | 0.1501 |
|  | More than 3 | None | 4.453028 | -1.11155 | 10.01761 | 0.1669 |
|  | 1 | More than 3 | 2.947324 | -3.92419 | 9.81884 | 0.6862 |
|  | 1 | 2 or 3 | 2.559631 | -4.63311 | 9.75238 | 0.7956 |
|  | 2 or 3 | More than 3 | 0.387693 | -5.33863 | 6.11402 | 0.9981 |
| **Bleeding** | Yes | Rarely/never | 0.811093 | -2.42223 | 4.044415 | 0.6223 |
| **Missing teeth** | None | 1 | 5.082393 | -1.2016 | 11.36636 | 0.1594 |
|  | More than 3 | 1 | 4.480636 | -5.9853 | 14.94662 | 0.6875 |
|  | 2 or 3 | 1 | 3.96478 | -5.5161 | 13.44564 | 0.7032 |
|  | None | 2 or 3 | 1.117613 | -7.1742 | 9.40942 | 0.9856 |
|  | None | More than 3 | 0.601758 | -8.8503 | 10.05379 | 0.9984 |
|  | More than 3 | 2 or 3 | 0.515855 | -11.0086 | 12.04033 | 0.9995 |
| **Toothache** | Sometimes | Previously, but not in the last 12 months | 3.505744 | -1.78383 | 8.795316 | 0.265 |
|  | Sometimes | Never | 2.019342 | -2.88761 | 6.926289 | 0.5979 |
|  | Never | Previously, but not in the last 12 months | 1.486403 | -2.99793 | 5.970732 | 0.716 |
| **Dry Mouth** | Yes, regularly | I don't know | 10.02659 | -0.01919 | 20.07237 | 0.0506 |
|  | No | I don't know | 8.59794 | 0.15261 | 17.04328 | 0.0442 |
|  | Yes, but infrequently | I don't know | 7.4263 | -1.35364 | 16.20623 | 0.1301 |
|  | Yes, regularly | Yes, but infrequently | 2.60029 | -4.84248 | 10.04307 | 0.8045 |
|  | Yes, regularly | No | 1.42865 | -5.41928 | 8.27657 | 0.9498 |
|  | No | Yes, but infrequently | 1.17165 | -3.83091 | 6.1742 | 0.9309 |
| **Bad breath** | Yes, regularly | Don't answer | 4.293339 | -6.36837 | 14.95505 | 0.7272 |
|  | Yes, regularly | No | 2.682397 | -5.59685 | 10.96164 | 0.8377 |
|  | Yes, regularly | Yes, but infrequently | 2.531694 | -5.18406 | 10.24745 | 0.8325 |
|  | Yes, but infrequently | Don't answer | 1.761644 | -6.62903 | 10.15232 | 0.9489 |
|  | No | Don't answer | 1.610942 | -7.27837 | 10.50025 | 0.9662 |
|  | Yes, but infrequently | No | 0.150703 | -4.73485 | 5.03626 | 0.9998 |

**Supplementary Table 29**– sweet liking and Oral health outcomes adjusted for age, sex, and smoking status.

| **Oral Health Outcome** | **Level** | **Differences*** | | | **Least Sq Mean** |
| --- | --- | --- | --- | --- | --- |
| **Caries** | More than 3 | A |  |  | 66.1662 |
|  | 1 | A |  |  | 66.13029 |
|  | 2 or 3 | A |  |  | 62.91062 |
|  | None | A |  |  | 62.08238 |
| **Bleeding** | Yes | A |  |  | 66.63485 |
|  | Rarely/never |  | B |  | 62.02836 |
| **Missing teeth** | 2 or 3 | A |  |  | 66.40599 |
|  | None | A |  |  | 65.23163 |
|  | 1 | A |  |  | 63.19071 |
|  | More than 3 | A |  |  | 59.72198 |
| **Toothache** | Previously, but not in the last 12 months | A |  |  | 67.07 |
|  | Sometimes | A | B |  | 64.17251 |
|  | Never |  | B |  | 62.50858 |
| **Dry Mouth** | I don't know | A |  |  | 67.80367 |
|  | Yes, but infrequently | A |  |  | 65.61323 |
|  | Yes, regularly | A |  |  | 65.17199 |
|  | No | A |  |  | 63.24683 |
| **Bad breath** | Yes, but infrequently | A |  |  | 66.30868 |
|  | Yes, regularly | A |  |  | 65.90045 |
|  | No | A |  |  | 63.18083 |
|  | Don't answer | A |  |  | 61.7362 |

*Levels not linked by the same letter are significantly difference at P<0.05 (see supplementary table 30 for p-values)

**Supplementary Table 30** – p-values, differences and 95% confidence intervals sweet liking and Oral health outcomes adjusted for age, sex, and smoking status.

| Oral Health Outcome | **Level 1** | **Level 2** | **Difference** | **Lower CL** | **Upper CL** | **p-Value** |
| --- | --- | --- | --- | --- | --- | --- |
| **Caries** | More than 3 | None | 4.083827 | -1.49071 | 9.65836 | 0.2343 |
|  | 1 | None | 4.047916 | -3.03592 | 11.13175 | 0.4547 |
|  | More than 3 | 2 or 3 | 3.255589 | -2.47315 | 8.98432 | 0.4596 |
|  | 1 | 2 or 3 | 3.219678 | -4.08477 | 10.52412 | 0.6673 |
|  | 2 or 3 | None | 0.828238 | -5.15267 | 6.80915 | 0.9844 |
|  | More than 3 | 1 | 0.035911 | -6.90823 | 6.98005 | 1 |
| **Bleeding** | Yes | Rarely/never | 4.606492 | 1.387833 | 7.825151 | 0.0051 |
| **Missing teeth** | 2 or 3 | More than 3 | 6.68401 | -4.83172 | 18.19974 | 0.4406 |
|  | None | More than 3 | 5.509644 | -3.6831 | 14.70239 | 0.4115 |
|  | 1 | More than 3 | 3.468728 | -6.95333 | 13.89078 | 0.8265 |
|  | 2 or 3 | 1 | 3.215283 | -6.30445 | 12.73502 | 0.8201 |
|  | None | 1 | 2.040917 | -4.29901 | 8.38084 | 0.8403 |
|  | 2 or 3 | None | 1.174366 | -7.11829 | 9.46702 | 0.9834 |
| **Toothache** | Previously, but not in the last 12 months | Never | 4.561417 | 0.03166 | 9.091175 | 0.048 |
|  | Previously, but not in the last 12 months | Sometimes | 2.89749 | -2.38078 | 8.175764 | 0.4013 |
|  | Sometimes | Never | 1.663927 | -3.20004 | 6.527891 | 0.7006 |
| **Dry Mouth** | I don't know | No | 4.556835 | -3.93813 | 13.0518 | 0.5108 |
|  | I don't know | Yes, regularly | 2.631682 | -7.51189 | 12.77526 | 0.9088 |
|  | Yes, but infrequently | No | 2.366394 | -2.6726 | 7.40539 | 0.6205 |
|  | I don't know | Yes, but infrequently | 2.190441 | -6.72423 | 11.10511 | 0.9213 |
|  | Yes, regularly | No | 1.925153 | -5.00356 | 8.85387 | 0.8907 |
|  | Yes, but infrequently | Yes, regularly | 0.441241 | -7.09281 | 7.97529 | 0.9988 |
| **Bad breath** | Yes, but infrequently | Don't answer | 4.572476 | -3.84385 | 12.98881 | 0.4997 |
|  | Yes, regularly | Don't answer | 4.164253 | -6.50548 | 14.83398 | 0.746 |
|  | Yes, but infrequently | No | 3.127843 | -1.72691 | 7.9826 | 0.3457 |
|  | Yes, regularly | No | 2.719619 | -5.50651 | 10.94575 | 0.8293 |
|  | No | Don't answer | 1.444633 | -7.41804 | 10.30731 | 0.975 |
|  | Yes, but infrequently | Yes, regularly | 0.408223 | -7.3133 | 8.12975 | 0.9991 |

**Supplementary Table 31**– sweet liking and Oral health outcomes adjusted for age, sex, smoking status, income, education and dietary index

| **Oral Health Outcome** | **Level** | **Differences*** | | | **Least Sq Mean** |
| --- | --- | --- | --- | --- | --- |
| **Caries** | 1 | A |  |  | 96.04897 |
|  | More than 3 | A |  |  | 94.85765 |
|  | 2 or 3 | A |  |  | 91.953 |
|  | None | A |  |  | 91.66836 |
| **Bleeding** | Yes | A |  |  | 94.28839 |
|  | Rarely/never |  | B |  | 90.93661 |
| **Missing teeth** | 2 or 3 | A |  |  | 95.67516 |
|  | None | A |  |  | 93.61012 |
|  | 1 | A |  |  | 93.36223 |
|  | More than 3 | A |  |  | 87.86429 |
| **Toothache** | Previously, but not in the last 12 months | A |  |  | 97.22632 |
|  | Sometimes | A | B |  | 93.30552 |
|  | Never |  | B |  | 92.26864 |
| **Dry Mouth** | I don't know | A |  |  | 96.93038 |
|  | Yes, but infrequently | A |  |  | 93.75601 |
|  | Yes, regularly | A |  |  | 93.68178 |
|  | No | A |  |  | 91.57444 |
| **Bad breath** | Yes, but infrequently | A |  |  | 95.66903 |
|  | Yes, regularly | A |  |  | 94.06594 |
|  | No | A |  |  | 93.26073 |
|  | Don't answer | A |  |  | 92.08693 |

*Levels not linked by the same letter are significantly difference at P<0.05 (see supplementary table 32 for p-values, differences in 95% confidence intervals)

**Supplementary Table 32** – p-values, differences and 95% confidence intervals sweetliking and Oral health outcomes adjusted for age, sex, smoking status, income, education and dietary index

| Oral Health Outcome | **Level 1** | **Level 2** | **Difference** | **Lower CL** | **Upper CL** | **p-Value** |
| --- | --- | --- | --- | --- | --- | --- |
| **Caries** | 1 | None | 4.380616 | -2.49236 | 11.25359 | 0.3555 |
|  | 1 | 2 or 3 | 4.095975 | -2.98029 | 11.17224 | 0.443 |
|  | More than 3 | None | 3.189295 | -2.28517 | 8.66376 | 0.4372 |
|  | More than 3 | 2 or 3 | 2.904655 | -2.72893 | 8.53824 | 0.5448 |
|  | 1 | More than 3 | 1.19132 | -5.56891 | 7.95155 | 0.9688 |
|  | 2 or 3 | None | 0.284641 | -5.52327 | 6.09255 | 0.9993 |
| **Bleeding** | Yes | Rarely/never | 3.351785 | 0.198128 | 6.505442 | 0.0373 |
| **Missing teeth** | 2 or 3 | More than 3 | 7.810866 | -3.48636 | 19.10809 | 0.2831 |
|  | None | More than 3 | 5.745829 | -3.51982 | 15.01148 | 0.3804 |
|  | 1 | More than 3 | 5.497942 | -4.76167 | 15.75755 | 0.5116 |
|  | 2 or 3 | 1 | 2.312924 | -6.98099 | 11.60684 | 0.9185 |
|  | 2 or 3 | None | 2.065037 | -6.06327 | 10.19335 | 0.9138 |
|  | None | 1 | 0.247887 | -5.91217 | 6.40794 | 0.9996 |
| **Toothache** | Previously, but not in the last 12 months | Never | 4.957679 | 0.58734 | 9.328022 | 0.0215 |
|  | Previously, but not in the last 12 months | Sometimes | 3.920798 | -1.23432 | 9.075915 | 0.1747 |
|  | Sometimes | Never | 1.036881 | -3.74534 | 5.8191 | 0.8667 |
| **Dry Mouth** | I don't know | No | 5.355933 | -2.94991 | 13.66178 | 0.3449 |
|  | I don't know | Yes, regularly | 3.248596 | -6.63126 | 13.12845 | 0.8316 |
|  | I don't know | Yes, but infrequently | 3.174367 | -5.46056 | 11.80929 | 0.7791 |
|  | Yes, but infrequently | No | 2.181566 | -2.73836 | 7.10149 | 0.663 |
|  | Yes, regularly | No | 2.107337 | -4.62748 | 8.84216 | 0.8513 |
|  | Yes, but infrequently | Yes, regularly | 0.074229 | -7.24562 | 7.39408 | 1 |
| **Bad breath** | Yes, but infrequently | Don't answer | 3.582106 | -4.62397 | 11.78818 | 0.6741 |
|  | Yes, but infrequently | No | 2.408301 | -2.36977 | 7.18637 | 0.5638 |
|  | Yes, regularly | Don't answer | 1.979012 | -8.44814 | 12.40616 | 0.9615 |
|  | Yes, but infrequently | Yes, regularly | 1.603094 | -5.94291 | 9.1491 | 0.9472 |
|  | No | Don't answer | 1.173805 | -7.51994 | 9.86755 | 0.9855 |
|  | Yes, regularly | No | 0.805206 | -7.29189 | 8.90231 | 0.9941 |
